# Magnetically Recoverable Nanoparticulate Catalysts for Cross-Coupling Reactions: The Dendritic Support Influences the Catalytic Performance

**DOI:** 10.3390/nano11123345

**Published:** 2021-12-09

**Authors:** Nina V. Kuchkina, Svetlana A. Sorokina, Alexey V. Bykov, Mikhail G. Sulman, Lyudmila M. Bronstein, Zinaida B. Shifrina

**Affiliations:** 1 A.N. Nesmeyanov Institute of Organoelement Compounds, Russian Academy of Sciences, 28 Vavilov St., 119991 Moscow, Russia; n_firsova@yahoo.com (N.V.K.); sorok.svetlana@gmail.com (S.A.S.); 2 Department of Biotechnology and Chemistry, Tver State Technical University, 22 A. Nikitina St., 170026 Tver, Russia; bykovav@yandex.ru (A.V.B.); sulmanmikhail@yandex.ru (M.G.S.); 3 Department of Chemistry, Indiana University, 800 E. Kirkwood Av., Bloomington, IN 47405, USA; 4 Department of Physics, Faculty of Science, King Abdulaziz University, P.O. Box 80303, Jeddah 21589, Saudi Arabia

**Keywords:** magnetic silica, dendron, catalyst, palladium, copper-free Sonogashira coupling, Heck coupling

## Abstract

Carbon-carbon cross-coupling reactions are among the most important synthetic tools for the preparation of pharmaceuticals and bioactive compounds. However, these reactions are normally carried out using copper, phosphines, and/or amines, which are poisonous for pharmaceuticals. The use of nanocomposite catalysts holds promise for facilitating these reactions and making them more environmentally friendly. In the present work, the PEGylated (PEG stands for poly(ethylene glycol) pyridylphenylene dendrons immobilized on silica loaded with magnetic nanoparticles have been successfully employed for the stabilization of Pd^2+^ complexes and Pd nanoparticles. The catalyst developed showed excellent catalytic activity in copper-free Sonogashira and Heck cross-coupling reactions. The reactions proceeded smoothly in green solvents at low palladium loading, resulting in high yields of cross-coupling products (from 80% to 97%) within short reaction times. The presence of magnetic nanoparticles allows easy magnetic separation for repeated use without a noticeable decrease of catalytic activity due to the strong stabilization of Pd species by rigid and bulky dendritic ligands. The PEG dendron periphery makes the catalyst hydrophilic and better suited for green solvents. The minor drop in activity upon the catalyst reuse is explained by the formation of Pd nanoparticles from the Pd^2+^ species during the catalytic reaction. The magnetic separation and reuse of the nanocomposite catalyst reduces the cost of target products as well as energy and material consumption and diminishes residual contamination by the catalyst. These factors as well as the absence of copper in the catalyst makeup pave the way for future applications of such catalysts in cross-coupling reactions.

## 1. Introduction

Cross-coupling is a powerful synthetic tool for the preparation of valuable chemicals widely used in industry [1,2,3]. The use of cross-coupling reactions has enabled the design of novel biologically active compounds, expanding the possibilities of the pharmaceutical industry [4,5]. While the most success in medicinal chemistry has been achieved by the Suzuki–Miyaura reaction of organoboron based nucleophiles with aryl halides [6,7], the Sonogashira and Heck reactions open opportunities for the syntheses of a number of valuable compounds containing unsaturated carbon-carbon bonds, often asymmetric or multisubstituted, being a part of different antibiotics, hormonal drugs, natural bioactive compounds, etc. [8,9].

The Sonogashira reaction, generally performed in organic solvents with a stoichiometric amount of amine base, Cu(I) salts, and homogeneous Pd catalysts suffers from the poor yield of target products due to side reactions (homocoupling of acetylenes and formation of enynes [10,11,12]), unsustainable reaction conditions, and the difficulty of separation and recyclability of the catalyst [13,14,15]. Moreover, the use of the homogeneous catalyst often leads to contamination of the final product with palladium, which is unacceptable for pharmaceuticals when the palladium content should not exceed ppb values [16]. Recent improvements in the Sonogashira reaction include the use of environmentally friendly and non-toxic solvents, elimination of Cu and amine, and switching to stable and reusable heterogeneous catalysts [1,5]. The same trend is observed for the Heck reaction [17,18].

Among the recently developed heterogeneous catalysts for cross-coupling reactions there are various supported catalysts, including palladium loaded mesoporous silica [19,20,21,22,23], metal and graphene oxides [24,25,26], zeolites [27,28,29], and organic supports, such as polymers and dendrimers [30,31,32,33,34,35]. Dendrimers are one of the most versatile macromolecular platforms for the stabilization of catalytically active metal complexes or nanoparticles (NPs) because of their hyperbranched architecture [36]. Dendrimer-based catalysts have been successfully utilized in hydrogenation [37,38,39], oxygen evolution reaction [40], oxidation [41,42], and cross-coupling reactions [43,44,45,46].

The formation of hybrid organic-inorganic catalysts has been shown to be a promising approach for the development of inexpensive and effective heterogeneous catalysts [47,48,49,50]. In this case, the catalyst consists of a solid support covalently bound to organic molecules and complexes or NPs of transition metals stabilized by the organic moiety. This approach was applied to the construction of dendrimer-containing catalysts, which were utilized in various organic reactions, including cross-coupling, showing excellent stability and activity [36,37,47,49]. Besides the apparent benefits of heterogeneous catalysts, the immobilization on a solid support offers supplemental advantages, e.g., ease of scaling up, tuning the affinity to polar solvents, and making the catalysts cheaper due to smaller amounts of capping molecules in comparison with colloidal catalysts without a solid support. An additional advantage can be realized for magnetically recoverable catalysts, where effective magnetic separation after the reaction can be provided [51,52].

In the preceding work we developed a novel approach for the immobilization of rigid aromatic pyridylphenylene dendrons on silica containing magnetite NPs [46]. The pyridine groups effectively stabilized a palladium precursor, resulting in the formation of Pd^0^ and Pd^2+^ species in different ratios, depending on the dendron structure [44,46]. The catalysts developed demonstrated an impressive activity in the Suzuki reaction of aryl halides with phenylboronic acid under mild conditions. The magnetic component provided the easy separation and recyclability in five consequent catalytic cycles. A comparison of the second and third generation dendrons revealed a positive dendritic effect on the yield of the target product and the catalyst activity. However, these catalysts are too hydrophobic to carry out reactions in more environmentally friendly hydrophilic solvents including an aqueous medium.

As such, here we report a catalytic nanocomposite based on magnetic silica and functionalized with new pyridylphenylene dendrons bearing hydrophilic PEG (PEG stands for poly(ethylene glycol)) tails in the periphery. The presence of nitrogen containing heterocycles in dendrons provides coordination with Pd species, while the PEG periphery facilitates their effective stabilization and catalysis in aqueous or “green” media. This catalyst is efficient in copper-free Sonogashira and Heck cross-coupling reactions under sustainable reaction conditions and low catalyst loading.

## 2. Materials and Methods

### 2.1. Materials

Potassium carbonate (K_2_CO_3_, ≥99%), iron(III) nitrate nonahydrate (98%), silica gel (99%, 6 nm pores, 200–425 mesh), 3-(aminopropyl)triethoxysilane (98%, APTES), 4-dimethylaminopyridine (98%), N,N′-dicyclohexylcarbodiimide (99%), N,N,N′,N″,N″-pentamethyldiethylenetriamine (PMDETA) (99%), poly(ethylene glycol) methyl ether azide (mPEG-N_3_, average Mn = 1000), 3-ethynylbenzoic acid (95%), palladium(II) acetate (98%), 4-iodoanisole, 98%, 4-iodotoluene, 99%, 4-bromoanisole, 99%, 3-bromoanisole, ≥98%, 2-bromoanisole, 97%, 4-bromotoluene, 98%, 3-bromotoluene, 98%, 2-bromotoluene, 99%, 4-bromonitrobenzene, 99%, 2-bromopyridine, 99%, styrene, (stabilised) for synthesis, acrylonitrile ((stabilised with hydroquinone monomethyl ether) for synthesis), methyl acrylate (stabilised with hydroquinone monomethyl ether for synthesis) were obtained from Sigma-Aldrich (St. Louis, MO, USA) and used without purification. Piperidine, 99%, ReagentPlus^®^, DMF, 99.8%, Et_3_N, 99%, THF (99.7%), acetonitrile (CH_3_CN, 99.9%), toluene (99%), ethanol (EtOH, ≥99.8%), benzene (99.8%), chloroform (CHCl_3_, ≥99%), and anhydrous sodium sulphate (Na_2_SO_4_, ≥99%) were purchased from Panreac (St. Louis, MO, USA) and used as received. Ammonium hydroxide solution (25%) was purchased from Σtec (Moscow, Russia). Distilled water was purified with an Elsi-Aqua water purification system. Spectra/Por^®^ 7 Standard RC Pre-treated Dialysis Tubing (MWCO 2 kDa) was purchased from Spectrum Laboratories (Los Angeles, CA, USA). All atmosphere-sensitive reactions were carried out under argon using Schlenk techniques. Analytical thin layer chromatography (TLC) was performed on commercial Merck plates coated with silica gel F-254.

### 2.2. Synthetic Procedures

#### 2.2.1. Preparation of the Catalyst MS-G3-PEG-Pd(OAc)_2_

##### Synthesis of G3-PEG

**Figure 1:**
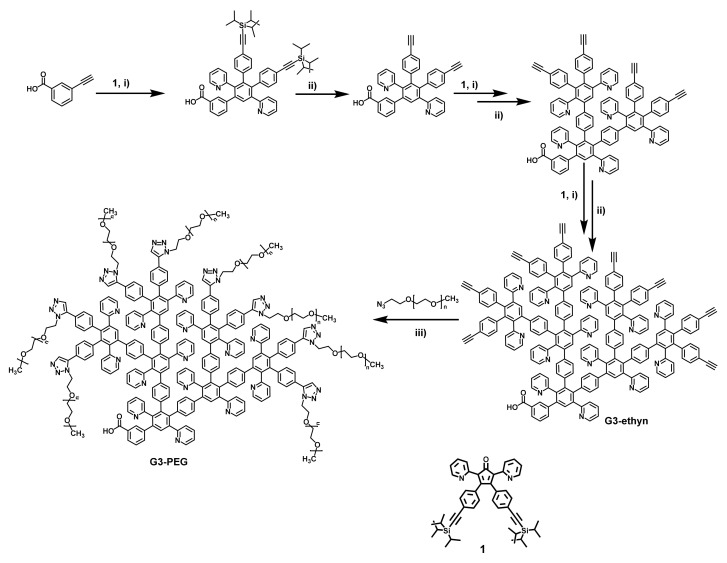
Synthesis of PEG functionalized pyridylphenylene dendron via Cu(I) catalyzed “click” reaction. Reaction conditions: (i) o-xylene, 145 °C; (ii) Bu_4_NF, THF, 25 °C; (iii) dry DMF, PMDETA, CuBr, 25 °C, 24 h.

The synthesis of G3-ethyn was carried out according to Figure 1 (see Results and Discussion section) and described in ref. [39].

In a typical experiment, G3-ethyn (0.1 g, 0.03 mmol), mPEG-N_3_ (0.44 g, 0.4 mmol), PMDETA (0.125 mL, 0.104 g, 0.6 mmol), and CuBr (0.09 g, 0.6 mmol) were stirred in 3.5 mL of dry DMF at 25 °C for 24 h in a Schlenk flask under argon. The reaction mixture was placed directly into a dialysis membrane (MWCO 2 kDa) and dialyzed against aqueous ammonia until the dialysate becomes colorless, then dialyzed against water until dialysate pH of 6–7 was reached. Yield: 86%. ^1^H NMR (400 MHz, CDCl_3_): 8.51–8.11 (m, 22H, PyH and H triazole ring), 7.7–6.5 (m, 109H, ArH), 4.57 (m, 16H, -CH_2_PEG), 3.90 (m, 16H, -CH_2_PEG), 3.64 (m, 736HPEG), 3.37 (m, 24H, -OMe-PEG). ICP analysis showed 85 ppb of Cu.

##### Synthesis of MS-NH_2_

The synthesis of MS (Fe_3_O_4_-SiO_2_) was carried out according to a procedure reported elsewhere [53]. The MS sample was functionalized with amino groups using APTES [44].

##### Attachment of G3-PEG to MS (MS-G3-PEG)

A synthesis of MS-G3-PEG was carried out using the formation of a peptide bond [44]. See the Supporting Information for details.

##### Preparation of MS-G3-PEG-Pd(OAc)_2_

Complexation of Pd (OAc)_2_ with MS-G3-PEG was carried out similar to the procedure reported elsewhere [44]. See the Supporting Information for details. Pd content (4.9%) was determined by XRF spectroscopy.

### 2.3. Testing MS-G3-PEG-Pd(OAc)_2_ in Cross-Coupling Reactions

#### 2.3.1. General Procedure for the Copper-Free Sonogashira Cross-Couplings

A round bottom flask (5 mL) equipped with a reflux condenser under aerobic conditions was charged with the aryl halide (1 mmol), base (2 mmol), MS-G3-PEG-Pd(OAc)_2_ (6.9–1.3 mg, 0.32–0.06 mol % of Pd with respect to aryl halide), and solvent (2 mL). Phenylacetylene (0.143 mL, 1.3 mmol) was added at 80 °C and the mixture was stirred for the required time (See Table 1). After completion of the reaction (monitored by GC at different time intervals), the reaction mixture was cooled to room temperature. The additional 2 mL of dichloromethane were added into the flask and the reaction solution was separated from the catalyst with a Pasteur pipette (Corning^®^, New York, NY, USA) while holding the catalyst with a rare-earth magnet at the flask wall. Then, the solution was poured into distilled water (50 mL), after which dichloromethane (40 mL) was added. The organic layer was separated, and the procedure was repeated until the pH of the aqueous phase became neutral. Then, the organic layer containing the coupling product was dried over anhydrous Na_2_SO_4_, filtered, and evaporated under reduced pressure. The products were isolated by silica gel column chromatography using hexane/dichloromethane mixture (4/1) as eluent. The ^1^H and ^13^C NMR data of isolated products are presented in the Supporting Information. The conversion, selectivity, and yield of the products are presented in Tables 1 and 2 (see “Results and Discussion” section).

The magnetic catalyst was washed in a reaction vessel with water, ethanol (to remove the traces of water), and dichloromethane (4 × 10 mL), using a rare-earth magnet for separation from solvents as discussed above. Then, it was dried at 70 °C to constant weight and reused in the same reaction vessel in the repeated coupling reactions under identical conditions.

#### 2.3.2. General Procedure for the Heck Cross-Couplings

A round bottom flask (5 mL) equipped with a reflux condenser under aerobic conditions was charged with aryl halide (1 mmol), K_2_CO_3_ (0.276 g, 2 mmol), MS-G3-PEG-Pd(OAc)_2_ (6.9–13 mg, 0.32–0.6 mol % of Pd with respect to aryl halide), and solvent (2.5 mL). Olefin (1.2 mmol) was added at 100 °C and the mixture was stirred for a required time (Table 3). After the completion of the reaction (monitored by GC at different time intervals), the reaction mixture was cooled to room temperature and poured into distilled water (50 mL). Then, dichloromethane (40 mL) was added to this mixture. The reaction mixture was treated similarly to the above procedure for Sonogashira coupling. The ^1^H and ^13^C NMR data of isolated products are presented in the Supporting Information. The conversion, selectivity, and yield of products are presented in Table 3 (see Results and Discussion section).

### 2.4. Characterization

^1^H and ^13^C NMR spectra were recorded on a Bruker Avance 400 (Bruker, Billerica, MA, USA). Chemical shifts are given in parts per million (ppm), using the solvent signal as a reference. CDCl_3_ was used as solvent for standard 1D NMR measurements (δ(1H) = 7.27 ppm, *δ*(13C) = 77.00 ppm).

Specimens for TEM, STEM, and EDXS studies were prepared by dipping the Lacey carbon film on Cu grid into the vail with the powder. The samples were studied using an Osiris TEM/STEM (Thermo Fisher Scientific, Waltham, MA, USA) equipped with a high-angle annular dark-field (HAADF) detector (Fischione, Corporate Circle Export, PA, USA) and a Super-X EDXS (Bruker, Billerica, MA, USA), at an accelerating voltage of 200 kV.

Powder X-ray diffraction (PXRD) patterns were collected using an Empyrean diffractometer (PANalytical). X-rays were generated from a copper target with a wavelength CuKα of 1.54187 Å. Soller slits, anti-scatter slits, divergence slits, and a nickel filter were in the beam path. During the measurement in reflection mode the sample was spinning with a revolution time of 4 s. The measurements were performed with various step-sizes and counting rates. The step-size for the experiments was 0.02 in the range 10–70 2*θ* degrees. The HighScore program was used for processing PXRD patterns for peak fitting.

X-ray photoelectron spectroscopy (XPS) data were obtained using Mg *Kα* (*hν* = 1253.6 eV) radiation with a ES-2403 spectrometer (Institute for Analytic Instrumentation of RAS, St. Petersburg, Russia) equipped with energy analyzer PHOIBOS 100-MCD5 (SPECS, Berlin, Germany) and X-ray source XR-50 (SPECS, Berlin, Germany). All the data were acquired at X-ray power of 250 W. Survey spectra were recorded at an energy step of 0.5 eV with an analyzer pass energy 40 eV, and high resolution spectra were recorded at an energy step of 0.05 eV with an analyzer pass energy 7 eV. Samples were allowed to outgas for 180 min before analysis and were stable during the examination. The data analysis was performed by CasaXPS.

The inductively coupled plasma optical emission spectroscopy (ICP-OES) (analyses were carried out using an Agilent ICP-OES5110 apparatus (Agilent, Santa Clara, CA, USA).

Gas chromatographic (GC) analyses were performed using a Chromatec-Crystal 5000.2 chromatograph (Chromatec, Yoshkar-Ola, Russia) equipped with a flame ionization detector (FID) and a DB-1 column (length = 100 m, inner diameter 0.25 mm and film thickness = 0.5 µm). The temperature program for the GC analysis was heated from 200 to 270 °C at 10°/min and held at 270 °C for 20 min. The inlet and detector temperatures were set at 295 and 290 °C, respectively. Sampling was carried out at time intervals of 10 min. Products were identified by comparison with authentic samples. For reliable identification and correlation between the GC signals and reaction products, the reaction mixture after catalytic reaction for each coupling partners was fractionated by column chromatography and the structure of isolated products was confirmed by NMR. These isolated samples were used as authentic samples for GC.

To obtain the Pd content from elemental analysis, X-ray fluorescence (XRF) measurements were performed with a Zeiss Jena VRA-30 spectrometer (Carl Zeiss Jena, Oberkochen, Germany) equipped with a Mo anode, a LiF200 crystal analyzer, and a SD detector. The time of data acquisition was held constant at 10 s. Analyses were based on the PdKα line, and a series of standards were prepared by mixing 1 g of polystyrene with 10–20 mg of standard compounds. Elemental analysis for C, H, and N was carried out using a Vario Microcube micro analyzer (Elementar, Langenselbold, Germany).

## 3. Results and Discussion

### 3.1. The Catalyst Synthesis and Characterization

The synthesis of the PEG functionalized third generation dendron is schematically presented in Figure 1. The binding of PEG units to the pyridylphenylene dendrons was carried out through the classic “click” reaction, i.e., Cu(I)-catalyzed azide-alkyne cycloaddition (CuAAC) of the methoxy polyethylene glycol azide (mPEG-N_3_) with alkyne terminated pyridylphenylene dendron (synthesized by us in the preceding work [39]) with the formation of a triazole cycle. During the synthesis, all terminal alkyne groups of the initial dendron were transformed into triazole groups with high yield, as is confirmed by the NMR analysis and MALDI ToF mass-spectrometry (Figures S1 and S2).

**Figure 2:**
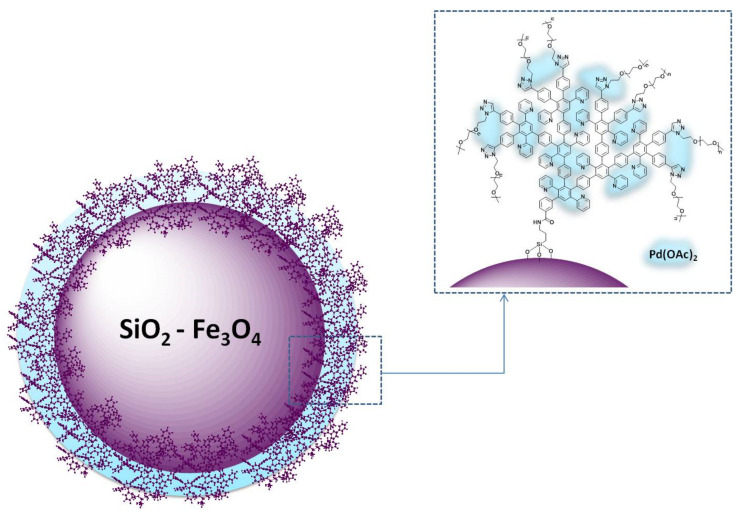
Schematic presentation of the catalytic composite MS G3-PEG-Pd(OAc)_2_.

For the development of the catalyst, we used an approach similar to that developed in our preceding work. This is based on the immobilization of the dendron on a magnetic silica surface via a reaction with amino functionalized silica [46], followed by complexation with palladium acetate [44,46]. The schematic image of the catalyst is presented in Figure 2.

**Figure 3:**
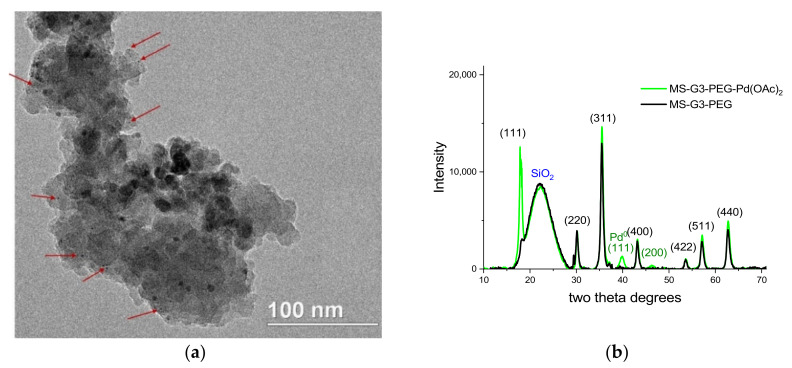
TEM image (**a**) and PXRD pattern (**b**) of MS-G3-PEG-Pd(OAc)_2_. Red arrows in (**a**) indicate small NPs with high electron density that are tentatively assigned to Pd NPs. Black notations for PXRD reflections in (**b**) are for spinel, while green notations are for Pd^0^.

The TEM image (Figure 3a) of MS-G3-PEG-Pd(OAc)_2_ shows a grey aggregate which represents magnetic silica along with NPs of higher electron density (indicated by red arrows in Figure 3a) which could be tentatively assigned to Pd NPs. Though no reducing agent was utilized in the catalyst fabrication, some reduction may occur due to reduction with CH_2_-NH-CO- groups, linking the dendron and the MS surface.

Figure 3b shows the PXRD patterns of MS-G3-PEG and MS-G3-PEG-Pd(OAc)_2_. Both patterns contain typical reflections characteristic of spinel (black notations) and a broad peak characteristic of amorphous silica. The Pd-containing sample also displays reflections at ~40 and 46 degrees two theta which can be attributed to (111) and (200) reflections of Pd^0^ (green notations), respectively. The disproportional increase of the (111) spinel reflection in the PXRD pattern of the catalyst compared to that of magnetic silica with attached dendrons was observed by us earlier for the analogous catalyst based on G3 dendrons without PEG periphery [47]. This change could be assigned to the formation of asymmetric spinel crystals upon Pd incorporation.

**Figure 4:**
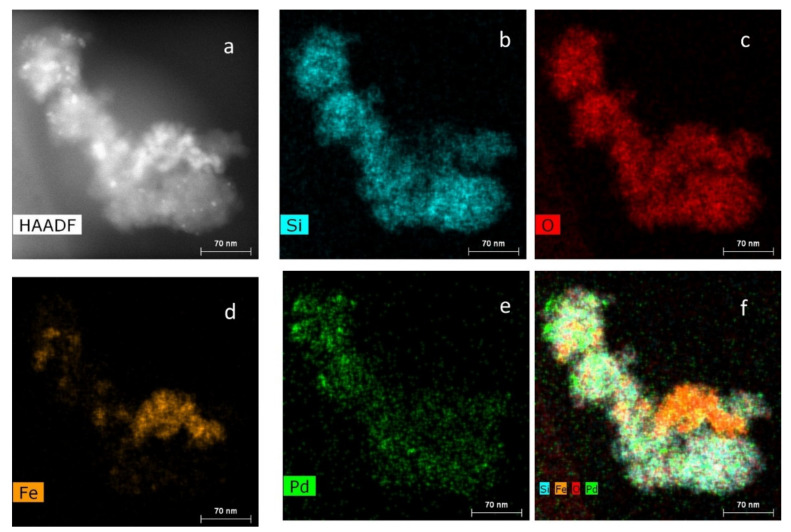
STEM dark-field image (**a**) and EDS maps of MS-G3-PEG-Pd(OAc)_2_ for Si (**b**), O (**c**), Fe (**d**), Pd (**e**), their superposition (**f**).

STEM EDS mapping, presented in Figure 4, was carried out to assess the location of elements in MS-G3-PEG-Pd(OAc)_2_. The Si (a) and O (b) maps are of the same shape as the piece of the material displayed in the STEM dark-field image (Figure 4a). The Fe map (d) demonstrates some aggregation of iron oxide NPs in the silica pores after the G3-PEG immobilization. Most probably this occurs due to poor compatibility of hydrophilic iron oxide NPs with the hydrophobic part of dendrons in silica pores. The Pd map (Figure 4e) shows two kinds of species: well dispersed Pd species across the piece of the catalyst and Pd NPs. The superposition of Si, O, Fe, and Pd maps is consistent with the above discussion. These data are in good agreement with TEM and PXRD.

**Figure 5:**
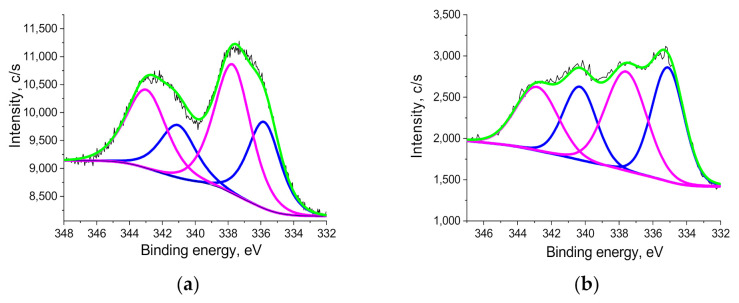
HR XPS Pd 3d of MS-G3-PEG-Pd(OAc)_2_ before (**a**) and after (**b**) the catalytic reaction. Black lines are the experimental data, the blue line is for Pd^0^, the magenta line is for Pd^2+^, and the green color is for the fitting curve.

To determine the Pd oxidation state, XPS was utilized. The high resolution (HR) XPS Pd 3d spectrum of MS-G3-PEG-Pd(OAc)_2_ is presented in Figure 5a. It was deconvoluted to represent two Pd species, Pd^0^ and Pd^2+^, with two peaks at 335.8 eV and 341.06 eV for the former and two peaks at 337.73 eV and 342.99 eV for the latter [54,55,56] (Table S1). Moreover, the peak positions for Pd^2+^ are consistent with those of Pd salts coordinated with nitrogen containing heterocycles [57,58,59]. The ratio of Pd^2+^:Pd^0^ is 1.68:1, indicating the prevalence of Pd^2+^ species.

The presence of dendrons on the magnetic silica surface was validated by the TGA analysis (Figure S3). The two-step character of the decomposition of MS-G3-PEG is observed in the TGA trace with the lower temperature region (from 250 to 400 °C) responsible for the decomposition of PEG tails with weight loss of around 25%. The higher temperature region (from 400 to 700 °C) can be assigned to the partial decomposition of the aromatic part of the dendron (15% weight loss) [44]. The high coke residue is due to the MS presence in the composite. A comparison of this TGA trace with that of parent magnetic silica, functionalized only with aminopropyltriethoxysilane linkers (MS-NH_2_) (Figure S3), allows for validation of the presence of the dendrons in MS-G3-PEG.

Thus, a combination of TEM, STEM, EDS, PXRD, XPS, and TGA clearly demonstrates that a magnetic silica aggregate is coated with PEGylated dendrons, bearing Pd complexes and NPs.

### 3.2. Catalytic Experiments

Initial experiments with 4-iodoanisole and phenylacetylene were performed in the mixture of H_2_O with EtOH (4:1) as solvent to optimize the reaction conditions such as base type, reaction temperature, and the Pd loading (see Table 1). The conversion into a coupled product was monitored by GC analysis. The products were characterized by the comparison of their GC signals with those of the authentic samples. As can be seen from Table 1 (entry 1), there was no product at 60 °C with K_2_CO_3_ as base after 6 h. Upon the reaction temperature increasing to 80 °C, a significant improvement was observed (conversion of 63.5 %, entry 2). The use of Et_3_N instead of K_2_CO_3_ increased the reaction rate significantly (Table 1, entry 3). However, the best results were obtained for piperidine instead of K_2_CO_3_, leading to the dramatic increase in the reaction rate and completion of the reaction for 30 min with 100% conversion and 98% selectivity (Table 1, entry 4). It is worth noting that an inert atmosphere was not beneficial as excellent conversion was also obtained in aerobic conditions (entry 5). Thus, all subsequent reactions were carried out under air.

The crucial effect of bases on Sonogashira coupling is well known. Beside their function as deprotonating agents, bases may substitute ligands to form more reactive complexes [60]. This advantage was maintained even at a decreased amount of Pd up to 0.12 mol % (Table 1, entries 5–7). However, a further decrease of the Pd loading to 0.06 mol % led to a drop of the conversion of 4-iodoanisole with poor selectivity regarding the target product (Table 1, entry 8). The kinetic curves presented in Figure S4a show an induction period in the reaction upon the decrease in the catalyst amount below 0.32 mol % Pd, indicating the formation of catalytic species during this time. It is noteworthy that, at 0.12 mol % Pd, 100% conversion can be reached for 3.5 h (Table 1, entry 7). We believe that at half of the Pd loading (0.06 mol %), the number of Pd atoms escaping the nanoparticle surface is very low and the reaction occurs slowly. When the reaction temperature is increased to 90 °C, the conversion reaches 100% but it requires 7 h.

A detailed analysis of the products of the cross-coupling reaction allowed us to identify by-products. It is worth noting that, despite the absence of Cu(I) as a co-catalyst, which induces Glaser-type oxidative homocoupling of terminal acetylenes [61], the formation of a negligible amount of 1,4-diphenylbuta-1,3-diyne occurs in the above reaction conditions, even in argon (Table 1, entries 2–7). The diyne was separated and confirmed by NMR (Figure S5). There are only a few papers where the formation of diynes as a side product in a copper-free Sonogashira coupling was revealed [62,63,64] and a few reports for the direct synthesis of diacetylenes using exclusively a Pd catalyst without a copper source [65,66,67]. In the majority of publications [68,69,70,71], no detection of diynes under copper-free conditions is reported. We assume this is because the focus of those papers was on an isolated yield of target products. However, our results are in good agreement with the results published earlier by Heck [62] as well as with a detailed analysis of the Sonogashira cross-coupling mechanism carried out by Kosmrlj [72], revealing the formation of small amounts of diynes in the copper-free Sonogashira reaction. Thus, to avoid the Glaser homocoupling, the 1.3 excess of phenylacetylene was employed, allowing us to reach high yields of target products. This choice was based on the kinetic data presented in the Supporting Information (Figure S4b).

The MS-D3-PEG-Pd(OAc)_2_ catalyst was further tested in Sonogashira cross-coupling reactions with other aryl halide substrates (Table 2, entries 2–10, Figure S6). As was expected, less-reactive bromides with electron-donating substituents required longer reaction times to give results comparable with those obtained for iodides (Table 2, entries 1–2, Figure S6). Nevertheless, the Sonogashira coupling proceeded quite efficiently at 0.12 mol % of Pd at 80 °C (Table 2, entries 4–10, Figure S6).

To further demonstrate the versatility of MS-G3-PEG-Pd(OAc)_2_ for cross-coupling reactions, we tested this catalyst in the Heck cross-coupling of various aryl halides with olefins (Table 3, Figure S7). The corresponding cross-coupling products were obtained for aryl halides bearing both electron-withdrawing and electron-donating groups with 93–97% yields. Typically, the Heck reaction requires harsher reaction conditions and prolonged reaction times [32,73]. We carried out the Heck reaction at the temperature of 100 °C, which is lower than usually employed, utilizing 0.6 mol % of Pd and K_2_CO_3_ as base in DMF. The reaction with less active aryl bromides with electron-donating groups proceeded at longer times, however high conversion and good selectivity were still attained without harshening of the reaction conditions.

### 3.3. Catalyst Recycling

**Figure 6:**
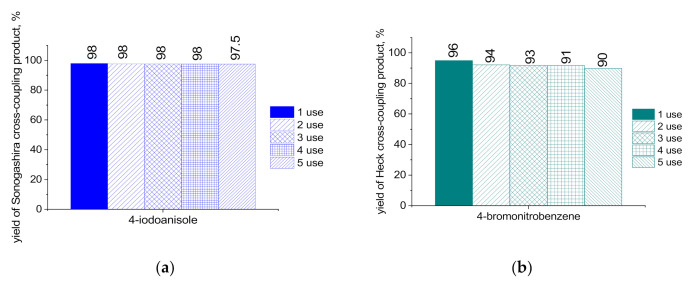
Yields of Sonogashira (**a**) and Heck (**b**) cross-coupling products in five consecutive runs with recovered catalysts.

The successful catalyst recovery and reuse are crucial aspects of catalytic processes as they determine their prospects for practical applications which require savings of materials and energy resources for economic benefits and the protection of the environment. The presence of magnetic NPs in the catalytic nanocomposite makes this procedure robust and efficient. We studied the recycling of MS-G3-PEG-Pd(OAc)_2_ in the Sonogashira and Heck cross-coupling reactions in several consecutive runs. The recycling tests were conducted for the Sonogashira reaction of 4-iodoanisole with phenylacetylene (Table 1, entry 5) as well as for the Heck reaction of 4-bromonitrobenzene with styrene (Table 3, entry 2). After each catalytic run, the catalyst was separated from the reaction mixture with an external magnet, washed, and reused in the same reactor. In case of the Sonogashira coupling, the MS-G3-PEG-Pd(OAc)_2_ catalyst maintained activity in five consecutive runs, with excellent preservation of the target product yields (Figure 6a). The same trend was observed for the Heck coupling reaction (Figure 6b).

**Figure 7:**
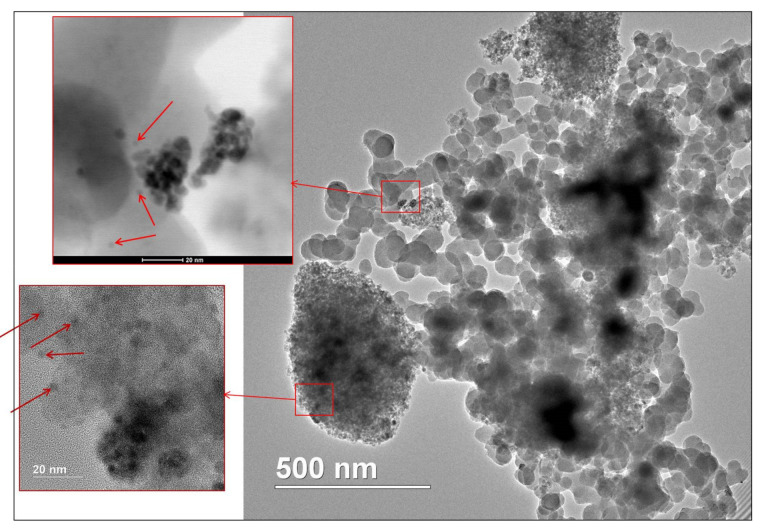
TEM image of MS-G3-PEG-Pd(OAc)_2_ after Sonogashira coupling (red arrows show Pd NPs).

At the same time, recycling is characterized by a noticeable drop of the reaction rate, which can be seen from the kinetic curves presented in Figures S8a,b and S9. The effect was more pronounced for the Heck reaction, which can be attributed to the harsher reaction conditions, favoring the contamination of the catalyst with reaction products. Nevertheless, a complete conversion of the substrates has been achieved, although requiring a longer time. To obtain further insight into this phenomenon, the magnetically recovered catalysts after the first run in the Sonogashira reaction of 4-iodoanisole with phenylacetylene and the Heck reaction of 4-bromonitrobenzene with styrene were examined by TEM, STEM EDS, and XPS. The TEM image of the catalyst after the Sonogashira coupling (Figure 7) shows the presence of small NPs (2.8 ± 0.4 nm in diameter) which are aggregated compared to those in initial MS-G3-PEG-Pd(OAc)_2_, while the NP size is preserved. A similar TEM image was observed after the first run of the Heck reaction (Figure S10).

**Figure 8:**
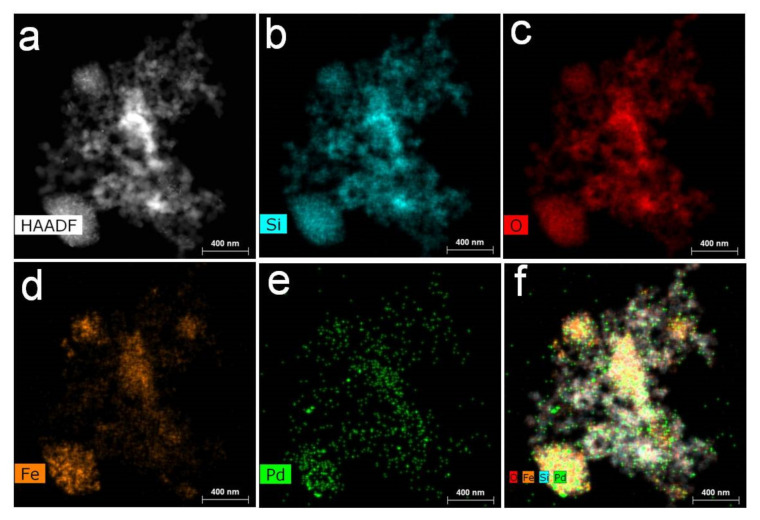
STEM dark-field image (**a**) and EDS maps for Si (**b**), O (**c**), Fe (**d**), and Pd (**e**) and their superposition for MS-G3-PEG-Pd after first catalytic cycle (**f**).

The STEM EDS mapping of this sample (Figure 8) confirms that these are Pd NPs located in the same space as Si, O, and Fe, i.e., on magnetic silica aggregates. The Pd map shows Pd species all over the material. The STEM EDS maps for MS-G3-PEG-Pd(OAc)_2_ after the first run of the Heck reaction look very similar (Figure S11).

The XPS Pd3d spectrum of MS-G3-PEG-Pd(OAc)_2_ after the first Sonogashira catalytic cycle is deconvoluted to the same Pd species as the initial catalyst (Figure 5b, Table S2), with the higher fraction of Pd^0^: the ratio of Pd^2+^:Pd^0^ is 1.1:1 while for the initial catalyst this ratio is 1.68:1.

Considering that both Sonogashira and Heck reactions are mainly utilized for syntheses of pharmaceuticals, the absence of Pd leaching is crucial for a promising catalyst. At the same time, we observed slower reactions in recycling which could be explained by a Pd loss. To clarify this issue, we used the ICP analysis and the hot catalytic test. The ICP analysis of the supernatant after the first and fifth catalytic cycles in Sonogashira coupling showed the presence of only 250 and 490 ppb of Pd, respectively. For the hot test, the catalyst was removed with an external magnet after 15 min of both Sonogashira and Heck reactions and the reaction was allowed to proceed for 3 h in another Schlenk flask. A subsequent GC analysis revealed no changes in conversion. The results of ICP and the hot test along with the XPS and STEM EDS data unambiguously demonstrate that there is no Pd leaching from the catalytic nanocomposite, while the decrease in the reaction rate should be associated with the formation of a greater fraction of Pd NPs during the catalytic reaction.

It is well known that a general mechanism of cross-coupling reactions involves the atomic Pd^0^ oxidative addition to an organic halide [72,74]. Therefore, the atomic Pd^0^ or Pd^2+^ containing catalysts are shown to be the most effective catalysts in cross-coupling reactions. In the case of nanoparticulate catalysts, the first catalytic step is believed to be the escape of metal atoms or clusters from the NP surface into a dendritic environment [75]. This is reflected in the longer reaction times, which is in good agreement with the decrease in the reaction rate of the recovered catalysts observed in this work. Nevertheless, the catalyst retained its excellent performance in terms of high yields of target products because the catalytic reaction occurs both on the Pd^2+^ species coordinated with dendron nitrogen containing heterocycles and on Pd atoms released from NPs. The difference in the catalytic behavior before and after the catalytic cycle concerns only the slope of the kinetic curve, reflecting changes in the reaction rate.

The results obtained confirm the strong stabilization of Pd species by pyridine and triazole groups of the PEGylated dendrons and the preservation of catalytic NPs in the dendritic environment. To prove the role of dendritic molecules, silica with magnetic NPs modified with 3-aminopropyltriethoxysilane but without dendrons was loaded with palladium acetate and tested in the Sonogashira reaction. The catalyst was removed from the reaction mixture and washed following the established procedure (see the Materials and Methods). The recycling experiment demonstrated a dramatic decrease in the catalyst activity (data not shown). The ICP analysis of the supernatant detected 5500 ppb of Pd, revealing an intense Pd leaching in the reaction mixture in the absence of dendritic ligands.

For comparison, the results reported in the literature over palladium-based catalysts are presented in Table 4 along with the data obtained in this work (Table 4, entries 10 and 17). The data show that the catalytic efficiencies of MS-G3-PEG-Pd(OAc)_2_ in the C-C bond formation via Sonogashira and Heck coupling reactions are close to or surpass those for the best heterogenous catalysts listed in Table 4 when comparing the reaction conditions, catalyst loading, and the yield of the target products. Additional advantages of the approach suggested here include (i) morphological stability of the catalyst, (ii) robust removal of the catalyst by an external magnet, and (iii) preservation of the yield of the target products after several catalytic runs. We believe the improved catalytic performance of dendritic catalysts is attributed to the special dendritic structure, containing long PEG tails to improve catalyst affinity to hydrophilic media and a rigid pyridylphenylene interior which improves the stabilization of the metal species, preventing their loss and contamination of the reaction products.

## 4. Conclusions

We developed a novel, magnetically recoverable Pd catalyst based on magnetic silica whose surface was modified with PEGylated pyridylphenylene dendrons. The PEGylated exterior of the dendron makes the catalyst more hydrophilic and provides excellent stabilization of Pd^2+^ species and Pd NPs along with the contribution from the pyridylphenylene part. This catalyst exhibits high activity and selectivity in copper, amine, and phosphine-free Sonogashira and Heck reactions under air, allowing high yields of the target products. Recycling experiments demonstrated the stability of the catalyst performance within five catalytic cycles. The presence of a magnetic support decorated with PEG tails allows for facile catalyst separation within one minute and the utilization of “green” solvents, such as H_2_O and ethanol. The catalytic study presented here and a comparison with literature data reveal the importance of the specific dendritic framework which preserves the catalytically active species and enables high activity and selectivity upon repeated use. The open, three-dimensional dendritic structure facilitates the mass transfer, enhancing the reaction rate and allowing one to avoid the retention of the reacting and target molecules in the catalyst space, preventing catalyst deactivation and the formation of side products. The absence of Pd leaching paves the way for using such catalytic systems in syntheses of pharmaceuticals and bioactive compounds.

## Figures and Tables

**Table 1:**
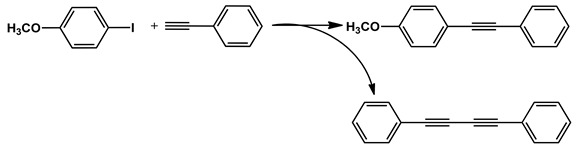
Reaction conditions of Sonogashira cross-coupling.

№ ^a^	Base	Pd Loading, mol %	T, °C	Time, h	Conversion, %	Selectivity, %	Yield ^c^, %	TOF, h^−1^ *
1	K_2_CO_3_	0.32	60	6	-	-	-	-
2	K_2_CO_3_	0.32	80	6	64	95	61	-
3	Et_3_N	0.32	80	1	62	98	60	-
4	Piperidine	0.32	80	0.5	100	98	98	-
5 ^b^	Piperidine	0.32	80	0.5	100	98	98	613
6 ^b^	Piperidine	0.2	80	2	100	98	98	731
7 ^b^	Piperidine	0.12	80	3.5	100	98	98	693
8 ^b^	Piperidine	0.06	80	4	2	76	2	-

Reaction conditions: ^a^ MS-G3-PEG-Pd(OAc)_2_ (6.9–1.3 mg, 0.32–0.06 mol % Pd), 4-iodoanisole/phenylacetylene (1 mmol/1.3 mmol), K_2_CO_3_ (2 mmol), H_2_O/EtOH (4/1, total 2.5 mL),argon, unless indicated otherwise; ^b^ air, ^c^ yield was calculated as multiplying the conversion by selectivity. Here and in Table 2 and Table 3 the conversion was calculated as a ratio of the area of the GC peak of the substrate to those of products and multiplied by 100%. The selectivity was calculated as a ratio of the area of the GC peak of the cross-coupling product to the peaks of all products obtained during the reaction (multiplied by 100%). * TOF is calculated considering the induction period for entries 6 and 7. The experimental reaction time used for the TOF calculation was the time, at which kinetic curves had a maximum slope (Figure S4). Thus, the induction period was subtracted from the reaction time.

**Table 2:**
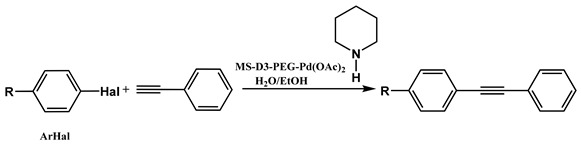
Sonogashira coupling of aryl halides with phenylacetylene.

№ ^a^	ArHal	Time, h	Conversion, %	Selectivity, %	Yield ^b^, %
1	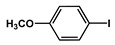	3.5	100	98	98
2	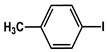	4.5	99	97	96
3	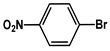	4	100	98	98
4	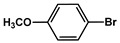	5	94	97	91
5	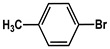	6	95	97	93
6	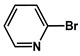	7	94	95	89
7	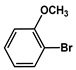	7	94	97	91
8	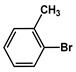	7	93	97	90
9	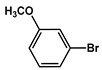	7	95	97	92
10	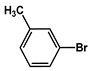	8	93	97	90

Reaction conditions: ^a^ MS-G3-PEG-Pd(OAc)_2_ (2.6 mg, 0.12 mol % Pd), ArHal/phenylacetylene (1 mmol/1.3 mmol), piperidine (2 mmol), H_2_O/EtOH (4/1, total 2.5 mL), 80 °C, air; ^b^ yield was calculated as multiplying conversion by selectivity.

**Table 3:**
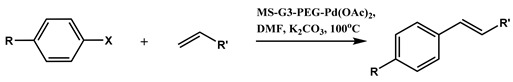
Heck cross-coupling reactions.

№ ^a^	ArHal	Olefin	Time, h.	Conversion, %	Selectivity, %	Yield ^c^, %
1 ^b^	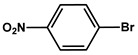	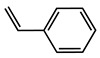	15	55	95	52
2			3 5	92 100	96 96	88 96
3	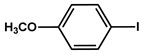		7	100	98	97
4	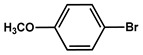		10	98	96	94
5	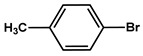		10	98	95	94
6	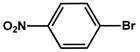	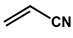	6	97	96	93
7	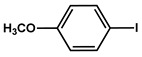		8	98	96	94
8	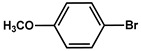		10	97	97	94
9	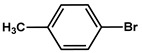		10	97	98	95
10	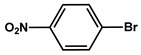	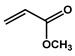	6	99	97	96
11	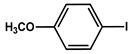		7	99	98	97
12	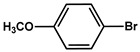		9	98	97	95
13	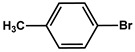		10	98	97	95

Reaction conditions: ^a^ MS-D3-PEG-Pd(OAc)_2_ (13 mg, 0.6 mol % Pd), ArHal/Olefin (1mmol/1.2 mmol), K_2_CO_3_ (2 mmol), DMF (2 mL), 100°C, air; ^b^ MS-D3-PEG-Pd(OAc)_2_ (6.9 mg, 0.32 mol % Pd); ^c^ yield was calculated as multiplying conversion by selectivity.

**Table 4:** Catalytic activity of MS-D3-PEG-Pd(OAc)_2_ in comparison with some other supported Pd catalysts used for Sonogashira (phenylacetylene with 4-iodoanisole) and Heck (4-bromonitrobenzene with styrene) coupling reactions.

Entry	Reaction	Catalyst	Conditions	Time (h)	Yield ^a^ (%)	Ref.
1	Sonogashira	Fe_3_O_4_@SiO_2_-NHC-Pd(II) (0.43 mol %)	Solvent-free, Piperidine, 90 °C	2.5	93	[71]
2	Sonogashira	Pd(II)Cl_2_–BTP@TMSP–nSiO_2_ (0.15 mol %)	H_2_O /DMF, DIPEA, 25 °C	2	95	[76]
3	Sonogashira	Pd-BIP-γ-Fe_2_O_3_@SiO_2_ (0.5 mol %)	DMF, Et_3_N, 100 °C	4	94	[77]
4	Sonogashira	Pd@TMU-16 (1.6 mol %)	EtOH, K_2_CO_3_, under reflux	0.5	93	[78]
5	Sonogashira	Pd NPs@CS-Kao(40 mg)	EtOH, K_2_CO_3_, 80 °C	3	85	[79]
6	Sonogashira	nSiO_2_-dendrimer-Pd(0) (0.085 mol %)	H_2_O, Et_3_N, 90 °C	2	91	[80]
7	Sonogashira	Pd@PTC-POP (0.3 mol %)	H_2_O, Et_3_N, 100 °C	2	96	[35]
8	Sonogashira	g-C_3_N_4_-Pd/CQDs@Fe (20 mg) (0.056 mol %)	H_2_O, K_2_CO_3_, 50 °C	0.75	80	[81]
9	Sonogashira	Fe_3_O_4_@SiO_2_–T/Pd (0.36 mol %)	DMF, Et_3_N, 110 °C	2.5	93	[82]
10	Sonogashira	MS-D3-PEG-Pd(OAc)_2_ (0.32 mol %) MS-D3-PEG-Pd(OAc)_2_ (0.12 mol %)	H_2_O/EtOH, Piperidine, 80 °C	0.5	93 98 ^b^	This work
				3.5	93 98 ^b^	
11	Heck	Pd-BIP-g-Fe_2_O_3_@SiO_2_ (0.5 mol %)	DMF, Et_3_N, 100 °C	3	93	[77]
12	Heck	G3-Gu-Pd (0.8 mol %)	H_2_O, SDS, K_2_CO_3_, 100 °C	12	90	[68]
13	Heck	MNPs-Mel-Pd (1.24 mol %)	DMSO, Et_3_N, 100 °C	3.5	81	[83]
14	Heck	Pd-NHC-MIL-101(Cr) catalyst (0.8 mol %)	DMF, K_2_CO_3_, 110 °C	8	95	[84]
15	Heck	MNPs-Mel-Pd (20 mg, 3.6 mol%)	DMF, K_2_CO_3_, 100 °C	8	86	[85]
16	Heck	MNPs-TDAH−Pd (30 mg, 5.9 mol %)	DMF, K_2_CO_3_, 100 °C	8	80	[86]
17	Heck	MS-G3-PEG-Pd(OAc)_2_ (0.6 mol %)	DMF, K_2_CO_3_, 100 °C	5	91 96 ^b^	This work

^a^ Isolated yields, unless otherwise stated; ^b^ Yield was calculated as multiplying the conversion by selectivity.

## Data Availability

The data presented in this study are available upon request from the corresponding author.

## Supplementary Materials

The following items are available online at https://www.mdpi.com/article/10.3390/nano11123345/s1, Figure S1: ^1^H NMR of PEGylated G3, Figure S2: MALDI ToF of PEGylated G3, Figure S3: TGA of MS-NH_2_ and MS-G3-PEG, Figure S4: Effect of the Pd loading (a) and the phenylacetylene molar ratio at the Pd loading of 0.32 mol % (b) on the reaction conversion, Figure S5: ^1^H NMR and ^13^C NMR of 1,4-diphenylbuta-1,3-diyne, Figure S6: Kinetic curves for different substrates at Pd loading of 0.12 mol %, Figure S7: Kinetic curves for Heck coupling of different substrates: (a) 4-bromonitrobenzene, (b) 4-iodoanisole, (c) 4-bromoanisole, (d) 4-bromotoluene, Figure S8: Kinetic curves for five consecutive catalytic cycles for Sonogashira coupling of 4-iodoanisole with phenylacetylene. In all cases, Pd loading is 0.32 mol % with respect to 4-iodoanisole, Figure S9: Kinetic curves for five consecutive catalytic cycles for Heck coupling of 4-bromonitrobenzene with styrene. In all cases, Pd loading is 0.6 mol % with respect to 4-bromonitrobenzene, Figure S10: TEM image of MS-G3-PEG-Pd(OAc)_2_ after the first cycle of Heck coupling¸ Figure S11: STEM dark-field image (a) and EDS maps for Si (b), O (c), Fe (d), and Pd (e) and their superposition (f) for MS-G3-PEG-Pd(OAc)_2_ after the first catalytic cycle of Heck coupling, Table S1: Fitting parameters for HR XPS Pd3d of MS-G3-PEG-Pd(OAc)_2_, Table S2: Fitting parameters for HR XPS Pd3d of MS-G3-PEG-Pd(OAc)_2_ after the first catalytic reaction.

## References

[1] Campeau L.C., Hazari N. (2019). Cross-Coupling and Related Reactions: Connecting Past Success to the Development of New Reactions for the Future. Organometallics.

[2] Gildner P.G., Colacot T.J. (2015). Reactions of the 21st Century: Two Decades of Innovative Catalyst Design for Palladium-Catalyzed Cross-Couplings. Organometallics.

[3] Snieckus V. (2016). New Trends in Cross-Coupling: Theory and Applications. Johns. Matthey’s Int. J. Res. Explor. Sci. Technol. Ind. Appl..

[4] Brow D.G., Bostrom J. (2016). Analysis of Past and Present Synthetic Methodologies on Medicinal Chemistry: Where Have All the New Reactions Gone?. J. Med. Chem..

[5] Magano J., Dunetz J.R. (2011). Large-Scale Applications of Transition Metal-Catalyzed Couplings for the Synthesis of Pharmaceuticals. Chem. Rev..

[6] Hooshmand S.E., Heidari B., Sedghi R., Varma R.S. (2019). Recent advances in the Suzuki-Miyaura cross-coupling reaction using efficient catalysts in eco-friendly media. Green Chem..

[7] Markovic T., Murray P.R.D., Rocke B.N., Shavnya A., Blakemore D.C., Willis M.C. (2018). Heterocyclic Allylsulfones as Latent Heteroaryl Nucleophiles in Palladium-Catalyzed Cross-Coupling Reactions. J. Am. Chem. Soc..

[8] Jagtap S. (2017). Heck Reaction-State of the Art. Catalysts.

[9] Albano G., Aronica L.A. (2020). Acyl Sonogashira Cross-Coupling: State of the Art and Application to the Synthesis of Heterocyclic Compounds. Catalysts.

[10] Ljungdahl T., Bennur T., Dallas A., Emtenas H., Martensson J. (2008). Two competing mechanisms for the copper-free Sonogashira cross-coupling reaction. Organometallics.

[11] Kurokhtina A.A., Larina E.V., Yarosh E.V., Schmidt A.F. (2016). Kinetic investigation of cross-coupling reaction steps by advanced competing reaction methods. J. Mol. Catal. A-Chem..

[12] Fantoni T., Bernardoni S., Mattellone A., Martelli G., Ferrazzano L., Cantelmi P., Corbisiero D., Tolomelli A., Cabri W., Vacondio F. (2021). Palladium Catalyst Recycling for Heck-Cassar-Sonogashira Cross-Coupling Reactions in Green Solvent/Base Blend. ChemSusChem.

[13] Gonzalez-Sebastian L., Morales-Morales D. (2019). Cross-coupling reactions catalysed by palladium pincer complexes. A review of recent advances. J. Organomet. Chem..

[14] Vasquez-Cespedes S., Betori R.C., Cismesia M.A., Kirsch J.K., Yang Q. (2021). Heterogeneous Catalysis for Cross-Coupling Reactions: An Underutilized Powerful and Sustainable Tool in the Fine Chemical Industry?. Org. Process Res. Dev..

[15] Pagliaro M., Pandarus V., Ciriminna R., Beland F., Cara P.D. (2012). Heterogeneous versus Homogeneous Palladium Catalysts for Cross-Coupling Reactions. ChemCatChem.

[16] Recho J., Black R.J.G., North C., Ward J.E., Wilkes R.D. (2014). Statistical DoE Approach to the Removal of Palladium from Active Pharmaceutical Ingredients (APIs) by Functionalized Silica Adsorbents. Org. Process Res. Dev..

[17] Arpad M. (2011). Efficient, Selective, and Recyclable Palladium Catalysts in Carbon-Carbon Coupling Reactions. Chem. Rev..

[18] Christoffel F., Ward T.R. (2018). Palladium-Catalyzed Heck Cross-Coupling Reactions in Water: A Comprehensive Review. Catal. Lett..

[19] Shevchuk M., Wang Q., Pajkert R., Xu J.C., Mei H.B., Roschenthaler G.V., Han J.L. (2021). Recent Advances in Synthesis of Difluoromethylene Phosphonates for Biological Applications. Adv. Synth. Catal..

[20] Andrade M.A., Martins L.M.D.R.S. (2020). New Trends in C–C Cross-Coupling Reactions: The Use of Unconventional Conditions. Molecules.

[21] Jacobs E., Keaveney S.T. (2021). Experimental and Computational Studies towards Chemoselective C–F over C–Cl Functionalisation: Reversible Oxidative Addition is the Key. ChemCatChem.

[22] Li P.H., Wang L., Zhang L., Wang G.W. (2012). Magnetic Nanoparticles-Supported Palladium: A Highly Efficient and Reusable Catalyst for the Suzuki, Sonogashira, and Heck Reactions. Adv. Synth. Catal..

[23] Das T., Uyama H., Nandi M. (2018). Pd-bound functionalized mesoporous silica as active catalyst for Suzuki coupling reaction: Effect of OAc^−^, PPh3 and Cl^−^ ligands on catalytic activity. J. Solid State Chem..

[24] Chua C.K., Sofer Z., Pumera M. (2016). Functionalization of Hydrogenated Graphene: Transition-Metal-Catalyzed Cross-Coupling Reactions of Allylic C–H Bonds. Angew. Chem.-Int. Ed..

[25] Diyarbakir S., Can H., Metin O. (2015). Reduced Graphene Oxide-Supported CuPd Alloy Nanoparticles as Efficient Catalysts for the Sonogashira Cross-Coupling Reactions. ACS Appl. Mater. Interfaces.

[26] Kim S., Jee S., Choi K.M., Shin D.S. (2021). Single-atom Pd catalyst anchored on Zr-based metal-organic polyhedra for Suzuki-Miyaura cross coupling reactions in aqueous media. Nano Res..

[27] Wang Y.Y., Liao J.P., Xie Z.Y., Zhang K., Wu Y., Zuo P., Zhang W.Q., Li J.Y., Gao Z.W. (2020). Zeolite-Enhanced Sustainable Pd-Catalyzed C–C Cross-Coupling Reaction: Controlled Release and Capture of Palladium. ACS Appl. Mater. Interfaces.

[28] Kumbhar A. (2017). Palladium Catalyst Supported on Zeolite for Cross-coupling Reactions: An Overview of Recent Advances. Top. Curr. Chem..

[29] Sain S., Kishore D., Jain S., Sharma V., Srivastava M., Sankararamakrishnan N., Mishra S., Dwivedi J., Wabaidur S.M., Sharma S. (2020). Zeolite enslaved transition metal complexes as novel heterogeneous catalysts for synthesis of polycyclic heterocycles using suzuki-miyaura cross coupling reaction under greener conditions. Inorg. Chem. Commun..

[30] Balinge K.R., Bhagat P.R. (2019). A polymer-supported salen-palladium complex as a heterogeneous catalyst for the Mizoroki-Heck cross-coupling reaction. Inorg. Chim. Acta.

[31] Wang Z.Z., Reddy C.B., Zhou X., Ibrahim J.J., Yang Y. (2020). Phosphine-Built-in Porous Organic Cage for Stabilization and Boosting the Catalytic Performance of Palladium Nanoparticles in Cross-Coupling of Aryl Halides. ACS Appl. Mater. Interfaces.

[32] Ma R., Yang P.B., Bian F.L. (2018). Magnetic dendritic polymer nanocomposites as supports for palladium: A highly efficient and reusable catalyst for Mizoroki-Heck and Suzuki-Miyaura coupling reactions. New J. Chem..

[33] Lu F., Astruc D. (2015). Catalytically Active Palladium Nanoparticle-Cored Ferrocenyl-Terminated Dendrimers. Eur J. Inorg Chem..

[34] Deraedt C., Wang D., Salmon L., Etienne L., Labrugere C., Ruiz J., Astruc D. (2015). Robust, Efficient, and Recyclable Catalysts from the Impregnation of Preformed Dendrimers Containing Palladium Nanoparticles on a Magnetic Support. ChemCatChem.

[35] Dong Y., Chen Y.Q., Jv J.J., Li Y., Li W.H., Dong Y.B. (2019). Porous organic polymer with in situ generated palladium nanoparticles as a phase-transfer catalyst for Sonogashira cross-coupling reaction in water. Rsc Adv..

[36] Bronstein L.M., Shifrina Z.B. (2011). Dendrimers as Encapsulating, Stabilizing, or Directing Agents for Inorganic Nanoparticles. Chem. Rev..

[37] Karakhanov E., Maximov A., Zolotukhina A., Mamadli A., Vutolkina A., Ivanov A. (2017). Dendrimer-Stabilized Ru Nanoparticles Immobilized in Organo-Silica Materials for Hydrogenation of Phenols. Catalysts.

[38] Wang W.J., Chamkina E.S., Cal E.G., Di Silvio D., Moro M.M., Moya S., Hamon J.R., Astruc D., Shifrina Z.B. (2021). Ferrocenyl-terminated polyphenylene-type “click” dendrimers as supports for efficient gold and palladium nanocatalysis. Dalton Trans..

[39] Kuchkina N.V., Yuzik-Klimova E.Y., Sorokina S.A., Peregudov A.S., Antonov D.Y., Gage S.H., Boris B.S., Nikoshvili L.Z., Sulman E.M., Morgan D.G. (2013). Polyphenylenepyridyl Dendrons with Functional Periphery and Focal Points: Syntheses and Applications. Macromolecules.

[40] Salmanion M., Najafpour M.M. (2021). Dendrimer-Ni-Based Material: Toward an Efficient Ni-Fe Layered Double Hydroxide for Oxygen-Evolution Reaction. Inorg. Chem..

[41] Kashani S.H., Landarani-Isfahani A., Moghadam M., Tangestaninejad S., Mirkhani V., Mohammadpoor-Baltork I. (2018). Nano-silica functionalized with thiol-based dendrimer as a host for gold nanoparticles: An efficient and reusable catalyst for chemoselective oxidation of alcohols. Appl. Organomet. Chem..

[42] Blanckenberg A., Kotze G., Swarts A.J., Malgas-Enus R. (2018). Effect of nanoparticle metal composition: Mono- and bimetallic gold/copper dendrimer stabilized nanoparticles as solvent-free styrene oxidation catalysts. J. Nanoparticle Res..

[43] Ricciardi R., Huskens J., Holtkamp M., Karst U., Verboom W. (2015). Dendrimer-Encapsulated Palladium Nanoparticles for Continuous-Flow Suzuki-Miyaura Cross-Coupling Reactions. ChemCatChem.

[44] Kuchkina N.V., Sorokina S.A., Lawson B.P., Torozova A.S., Nikoshvili L.Z., Sulman E.M., Lependina O.L., Stein B.D., Pink M., Morgan D.G. (2020). Dendritic effect for immobilized pyridylphenylene dendrons in hosting catalytic Pd species: Positive or negative?. React. Funct. Polym..

[45] Deraedt C., Salmon L., Astruc D. (2014). “Click” Dendrimer-Stabilized Palladium Nanoparticles as a Green Catalyst Down to Parts per Million for Efficient C–C Cross-Coupling Reactions and Reduction of 4-Nitrophenol. Adv. Synth. Catal..

[46] Sorokina S.A., Kuchkina N.V., Lawson B.P., Krasnova I.Y., Nemygina N.A., Nikoshvili L.Z., Talanova V.N., Stein B.D., Pink M., Morgan D.G. (2019). Pyridylphenylene dendrons immobilized on the surface of chemically modified magnetic silica as efficient stabilizing molecules of Pd species. Appl. Surf. Sci.

[47] Lee D.W., Yoo B.R. (2016). Advanced silica/polymer composites: Materials and applications. J. Ind. Eng. Chem..

[48] Wang Q., Zhang Y., Zhou Y., Zhang Z., Xu Y., Zhang C., Sheng X. (2015). Synthesis of dendrimer-templated Pt nanoparticles immobilized on mesoporous alumina for p-nitrophenol reduction. New J. Chem..

[49] Karakhanov E., Maximov A., Kardasheva Y., Semernina V., Zolotukhina A., Ivanov A., Abbott G., Rosenberg E., Vinokurov V. (2014). Pd Nanoparticles in Dendrimers Immobilized on Silica–Polyamine Composites as Catalysts for Selective Hydrogenation. ACS Appl. Mater. Interfaces.

[50] Giacalone F., Campisciano V., Calabrese C., La Parola V., Syrgiannis Z., Prato M., Gruttadauria M. (2016). Single-Walled Carbon Nanotube–Polyamidoamine Dendrimer Hybrids for Heterogeneous Catalysis. ACS Nano.

[51] Kazemi M. (2020). Based on magnetic nanoparticles: Gold reusable nanomagnetic catalysts in organic synthesis. Synth. Commun..

[52] Shifrina Z.B., Matveeva V.G., Bronstein L.M. (2020). Role of Polymer Structures in Catalysis by Transition Metal and Metal Oxide Nanoparticle Composites. Chem. Rev..

[53] Oracko T., Jaquish R., Losovyj Y.B., Morgan D.G., Pink M., Stein B.D., Doluda V.Y., Tkachenko O.P., Shifrina Z.B., Grigoriev M.E. (2017). Metal-Ion Distribution and Oxygen Vacancies That Determine the Activity of Magnetically Recoverable Catalysts in Methanol Synthesis. ACS Appl. Mater. Interfaces.

[54] Begum T., Mondal M., Borpuzari M.P., Kar R., Kalita G., Gogoi P.K., Bora U. (2017). An immobilized symmetrical bis-(NHC) palladium complex as a highly efficient and recyclable Suzuki-Miyaura catalyst in aerobic aqueous media. Dalton Trans..

[55] Lei P., Hedlund M., Lomoth R., Rensmo H., Johansson O., Hammarstrom L. (2008). The role of colloid formation in the photoinduced H_2_ production with a Ru^II^-Pd^II^ supramolecular complex: A study by GC, XPS, and TEM. J. Am. Chem. Soc..

[56] Wojnicki M., Socha R.P., Pcdzich Z., Mech K., Tokarski T., Fitzner K. (2018). Palladium(II) Chloride Complex Ion Recovery from Aqueous Solutions Using Adsorption on Activated Carbon. J. Chem. Eng Data.

[57] Mathew J.P., Srinivasan M. (1995). Photoelectron-Spectroscopy (Xps) Studies on Some Palladium Catalysts. Eur. Polym. J..

[58] Darlatt E., Traulsen C.H.H., Poppenberg J., Richter S., Kuhn J., Schalley C.A., Unger W.E.S. (2012). Evidence of click and coordination reactions on a self-assembled monolayer by synchrotron radiation based XPS and NEXAFS. J. Electron. Spectrosc..

[59] Poppenberg J., Richter S., Darlatt E., Traulsen C.H.H., Min H., Unger W.E.S., Schalley C.A. (2012). Successive coordination of palladium(II)-ions and terpyridine-ligands to a pyridyl-terminated self-assembled monolayer on gold. Surf. Sci..

[60] Tougerti A., Negri S., Jutand A. (2007). Mechanism of the copper-free palladium-catalyzed Sonagashira reactions: Multiple role of amines. Chem.-Eur. J..

[61] Kuchkina N.V., Haskell A.K., Sorokina S.A., Torozova A.S., Nikoshvili L.Z., Sulman E.M., Stein B.D., Morgan D.G., Bronstein L.M., Shifrina Z.B. (2020). Pd Catalyst Based on Hyperbranched Polypyridylphenylene Formed In Situ on Magnetic Silica Allows for Excellent Performance in Suzuki-Miyaura Reaction. ACS Appl. Mater. Interfaces.

[62] Dieck H.A., Heck F.R. (1975). Palladium Catalyzed Synthesis of Aryl, Heterocyclic and Vinylic Acetylene Derivatives. J. Organomet. Chem..

[63] Bandini M., Luque R., Budarin V., Macquarrie D.J. (2005). Aryl alkynylation versus alkyne homocoupling: Unprecedented selectivity switch in Cu, phosphine and solvent-free heterogeneous Pd-catalysed couplings. Tetrahedron.

[64] De Cattelle A., Billen A., O’Rourke G., Brullot W., Verbiest T., Koeckelberghs G. (2019). Ligand-free, recyclable palladium-functionalized magnetite nanoparticles as a catalyst in the Suzuki-, Sonogashira, and Stille reaction. J. Organomet. Chem..

[65] Atobe S., Sonoda M., Suzuki Y., Yamamoto T., Masuno H., Shinohara H., Ogawa A. (2013). Palladium-catalyzed oxidative homocoupling reaction of terminal acetylenes using trans-bidentaTable 1-(2-pyridylethynyl)-2-(2-thienylethynyl)benzene. Res. Chem. Intermediat..

[66] Ye C.F., Xiao X.C., Twamley B., LaLonde A.D., Norton M.G., Shreeve J.M. (2007). Basic ionic liquids: Facile solvents for carbon-carbon bond formation reactions and ready access to palladium nanoparticles. Eur. J. Org. Chem..

[67] Perrone S., Bona F., Troisi L. (2011). Palladium-catalyzed acylation and/or homo-coupling of aryl- and alkyl-acetylenes. Tetrahedron.

[68] Niknam E., Moaddeli A., Khalafi-Nezhad A. (2020). Palladium anchored on guanidine-terminated magnetic dendrimer (G3-Gu-Pd): An efficient nano-sized catalyst for phosphorous-free Mizoroki-Heck and copper-free Sonogashira couplings in water. J. Organomet. Chem..

[69] Gogoi R., Saikia R., Borah G. (2019). Agro waste derived nanosilica supported Pd(II) complex: A protocol for copper free Sonogashira reaction in water. J. Organomet. Chem..

[70] Nasrollahzadeh M., Khalaj M., Ehsani A. (2014). A heterogeneous and reusable nanopolymer-supported palladium catalyst for the copper- and phosphine-free Sonogashira coupling reaction under aerobic conditions in water. Tetrahedron Lett..

[71] Esmaeilpour M., Sardarian A.R., Firouzabadi H. (2018). N-heterocyclic carbene-Pd(II) complex based on theophylline supported on Fe_3_O_4_@SiO_2_ nanoparticles: Highly active, durable and magnetically separable catalyst for green Suzuki-Miyaura and Sonogashira-Hagihara coupling reactions. J. Organomet. Chem..

[72] Gazvoda M., Virant M., Pinter B., Kosmrlj J. (2018). Mechanism of copper-free Sonogashira reaction operates through palladium-palladium transmetallation. Nat. Commun..

[73] Wang D., Deraedt C., Salmon L., Labrugere C., Etienne L., Ruiz J., Astruc D. (2015). Efficient and Magnetically Recoverable “Click” PEGylated gamma-Fe_2_O_3_-Pd Nanoparticle Catalysts for Suzuki-Miyaura, Sonogashira, and Heck Reactions with Positive Dendritic Effects. Chem.-Eur. J..

[74] Ananikov V.P., Beletskaya I.P. (2012). Toward the Ideal Catalyst: From Atomic Centers to a “Cocktail” of Catalysts. Organometallics.

[75] Eremin D.B., Ananikov V.P. (2017). Understanding active species in catalytic transformations: From molecular catalysis to nanoparticles, leaching, “Cocktails” of catalysts and dynamic systems. Coord. Chem. Rev..

[76] Dehbanipour Z., Moghadam M., Tangestaninejad S., Mirkhani V., Mohammadpoor-Baltork I. (2017). Nano-silica supported palladium catalyst: Synthesis, characterization and application of its activity in Sonogashira cross-coupling reactions. J. Organomet. Chem..

[77] Sobhani S., Zeraatkar Z., Zarifi F. (2015). Pd complex of an NNN pincer ligand supported on γ-Fe_2_O_3_@SiO_2_ magnetic nanoparticles: A new catalyst for Heck, Suzuki and Sonogashira coupling reactions. New J. Chem..

[78] Kiani A., Alinezhad H., Ghasemi S. (2021). Versatile and an efficient Sonogashira coupling reaction catalyzed with modified Pd-functionalized TMU-16 as a novel and reusable nanocatalyst. J. Organomet. Chem..

[79] Nasrollahzadeh M., Shafiei N., Baran T., Pakzad K., Tahsili M.R., Baran N.Y., Shokouhimehr M. (2021). Facile synthesis of Pd nanoparticles supported on a novel Schiff base modified chitosan-kaolin: Antibacterial and catalytic activities in Sonogashira coupling reaction. J. Organomet. Chem..

[80] Esmaeilpour M., Sardarian A., Javidi J. (2016). Dendrimer-encapsulated Pd(0) nanoparticles immobilized on nanosilica as a highly active and recyclable catalyst for the copper- and phosphine-free Sonogashira–Hagihara coupling reactions in water. Catal. Sci. Technol..

[81] Mohammadi L., Heravi M.M., Sadjadi S., Malmir M. (2019). Hybrid of Graphitic Carbon Nitride and Palladated Magnetic Carbon Dot: An Efficient Catalyst for Coupling Reaction. ChemistrySelect.

[82] Elhampour A., Nemati F., Nahzomi H.T., Mohagheghi V. (2017). Magnetic nanoparticle-supported tetrazole-functionalized palladium catalyst: Synthesis, DFT study and application for Sonogashira and Heck cross-coupling reactions. Res. Chem. Intermediat..

[83] Aryanasab F., Shabanian M., Laoutid F., Vahabi H. (2021). Immobilizing palladium on melamine-functionalized magnetic nanoparticles: An efficient and reusable phosphine-free catalyst for Mizoroki–Heck reaction. Appl. Organomet. Chem..

[84] Niknam E., Panahi F., Khalafi-Nezhad A. (2021). Immobilized Pd on a NHC-functionalized metal-organic FrameworkMIL-101(Cr): An efficient heterogeneous catalyst in the heck and copper-free Sonogashira coupling reactions. J. Organomet. Chem..

[85] Bodaghifard M.A. (2019). Palladium-melamine complex anchored on magnetic nanoparticles: A novel promoter for C–C cross coupling reaction. J. Organomet. Chem..

[86] Asadbegi S., Bodaghifard M.A., Alimohammadi E., Ahangarani-Farahani R. (2018). Immobilization of Palladium on Modified Nanoparticles and Its Catalytic Properties on Mizoroki-Heck Reaction. ChemistrySelect.

