# Supplementary material for: Magnetically Recoverable Nanoparticulate Catalysts for Cross-Coupling Reactions: The Dendritic Support Influences the Catalytic Performance
Source: Nanomaterials (Basel). 2021 Dec 9;11(12):3345. doi: 10.3390/nano11123345 (PMC8708486; doi:10.3390/nano11123345)
Supplement: Supplementary file 1 [file nanomaterials-11-03345-s001.zip › nanomaterials-1475157-supplementary.pdf]

Supporting information

Magnetically Recoverable Nanoparticulate Catalysts for Cross-Coupling Reactions: The Dendritic Support Influences the Catalytic Performance

Nina V. Kuchkina [1], Svetlana A. Sorokina [1], Alexey V. Bykov [2], Mikhail G. Sulman [2], Lyudmila M. Bronstein [1,3,4,*] and Zinaida B. Shifrina [1,*]

[1] A.N. Nesmeyanov Institute of Organoelement Compounds, Russian Academy of Sciences, 28 Vavilov St., 119991 Moscow, Russia; n_firsova@yahoo.com (N.V.K.); sorok.svetlana@gmail.com (S.A.S.)
[2] Department of Biotechnology and Chemistry, Tver State Technical University, 22 A. Nikitina St., 170026 Tver, Russia; bykovav@yandex.ru (A.V.B.); sulmanmikhail@yandex.ru (M.G.S.)
[3] Department of Chemistry, Indiana University, 800 E. Kirkwood Av., Bloomington, IN 47405, USA
[4] Department of Physics, Faculty of Science, King Abdulaziz University, P.O. Box 80303, Jeddah 21589, Saudi Arabia
* Correspondence: lybronst@indiana.edu (L.M.B.); shifrina@ineos.ac.ru (Z.B.S.)

Experimental

Attachment of G3-PEG to MS (MS-G3-PEG):

The reaction was carried out under argon in a Schlenk flask, furnished with a reflux condenser and a mechanical stir bar. 4-Dimethylaminopyridine (2.7 mg, 0.02 mmol) was added to the solution of G3-PEG (306.6 mg, 0.026 mmol) in dichloromethane (1 mL). The reaction mixture was stirred for 10 min at room temperature and then 12.4 mg (0.06 mmol) of N,N′-dicyclohexylcarbodiimide dissolved in 0.2 mL of dichloromethane was added. The mixture was stirred at room temperature for additional 40 min. Then 110.8 mg of amino-functionalized magnetic silica MS-NH_2 was added slowly to the prepared mixture and stirred at 40 °C for 32 h. The product was collected from the suspension with a magnet and washed with dichloromethane (100 mL), ethanol (50 mL) and acetone (30 mL) and dried in vacuum. The elemental analysis data, %: C 41.00, N 3.76, H 4.72.

Complexation of Pd $(OAc)_2$ with MS-G3-PEG (MS-G3-PEG-Pd$(OAc)_2$):

The suspension of MS-G3-PEG (100 mg) in toluene (200 mL) was vigorously stirred and loaded with Pd$(OAc)_2$ (71.4 mg) in toluene (72 mL). The stirring continued

for 24 h at room temperature. The product was separated from the mixture with a magnet and thoroughly washed with toluene (100 mL), ethanol (100 mL) and dichloromethane (50 mL) and dried at room temperature in vacuum.

The compounds synthesized via Sonogashira and Heck reactions with MS-G3-PEG-Pd(OAc)₂:

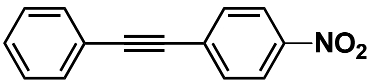

1-Nitro-4-(phenylethynyl)benzene: yellow solid ^{1}H NMR: δ = 8.22 (d, J = 8.7 Hz, 2H), 7.67 (d, J = 8.7 Hz, 2H), 7.57–7.55 (m, 2H), 7.41–7.38 (m, 3H) (ppm); ^{13}C NMR: δ = 146.9, 132.3, 131.9, 130.3, 129.3, 128.6, 123.6, 122.1, 94.7, 87.5 (ppm);

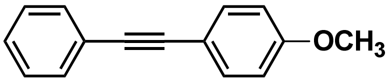

1-Methoxy-4-(phenylethynyl)benzene: white solid ^{1}H NMR: δ = 7.57–7.46 (m, 4H), 7.38–7.30 (m, 3H), 6.89 (d, J=8.6 Hz, 2H), 3.83 (s, 3H) (ppm); ^{13}C NMR: δ = 159.6, 133.0, 131.5, 128.3, 127.9, 123.6, 115.4, 114.0, 89.4, 88.1, 55.3 (ppm);

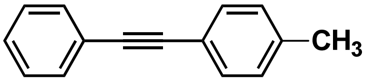

1-Methyl-4-(phenylethynyl)benzene: white solid^{1}H NMR: δ = 7.54–7.51 (m, 2H), 7.43 (d, J=8.1 Hz, 2H), 7.37–7.31 (m, 3H), 7.16 (d, J=7.8 Hz, 2H), 2.37 (s, 3H) (ppm); ^{13}C NMR: δ = 138.4, 132.5, 131.5, 129.1, 128.3, 128.1, 123.5, 120.2, 89.5, 88.7, 21.5 (ppm);

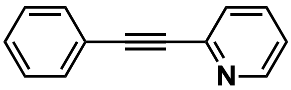

2-(Phenylethynyl)pyridine: brown oil; ^{1}H NMR: δ = 8.55 (d, 1H, J = 5 Hz), 7.58–7.63 (m, 1H), 7.51–7.55 (m, 2H), 7.46 (d, 1H, J = 7 Hz), 7.26–7.35 (m, 3H), 7.28–7.23 (m, 1H) (ppm); ^{13}C NMR: δ = 149.7, 143.2, 136.5, 132.1, 129.1, 128.4, 127.2, 122.8, 122.2, 89.9, 88.2 (ppm);

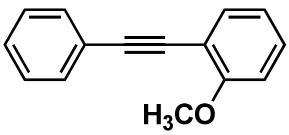

1-Methoxy-2-(2-phenylethynyl)benzene: pale yellow oil; ^{1}H NMR: δ = 7.58-7.54 (m, 2H), 7.50-7.53 (m, 1H), 7.36-7.28 (m, 4H), 6.94-6.91 (m, 1H), 6.90 (d, J = 7.9 Hz, 1H), 3.91 (s, 3H) (ppm); ^{13}C NMR: δ = 159.9, 133.6, 131.6, 129.7, 128.2, 128.1, 123.5, 120.4, 112.4, 110.6, 93.4, 85.7, 55.8 (ppm);

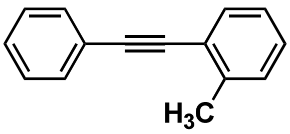

1-Methyl-2-(2-phenylethynyl)benzene: colorless oil; ^{1}H NMR: δ = 7.62-7.58 (m, 3H), 7.43-7.37 (m, 3H), 7.30-7.29 (m, 2H), 7.25-7.21 (m, 1H), 2.59 (s, 3H) (ppm). ^{13}C NMR: δ = 140.17, 131.84, 131.51, 129.46, 128.35, 128.30, 128.16, 125.58, 123.58, 123.05, 93.37, 88.37, 20.73 (ppm).

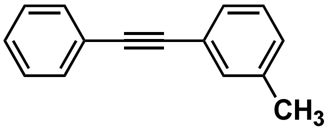

1-Methyl-3-(2-phenylethynyl)benzene: yellow solid; ^{1}H NMR: δ = 7.58-7.55 (m, 2 H), 7.40-7.36 (m, 5 H), 7.28-7.25 (m, 1 H), 7.18-7.17 (m, 1 H), 2.38 (s, 3 H) (ppm). ^{13}C NMR: δ = 138.0, 132.2, 131.6, 129.2, 128.7, 128.3, 128.3, 128.2, 123.4, 123.1, 89.6, 89.1, 21.3 (ppm).

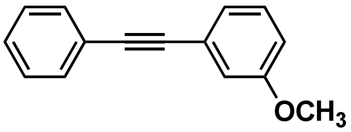

1-Methoxy-3-(2-phenylethynyl)benzene: yellow solid; ^{1}H NMR: δ = 7.62-7.60 (m, 2H), 7.43-7.39 (m, 3H), 7.33-7.32 (m, 1H), 7.22-7.20 (d, 1H), 7.14 (s, 1H), 6.97-6.94 (m, 1H), 3.86 (s, 3H) (ppm); ^{13}C NMR: δ = 159.43, 131.70, 129.49, 128.43, 124.33, 124.25, 123.26, 116.42, 115.00, 89.42, 89.29, 55.30 (ppm).

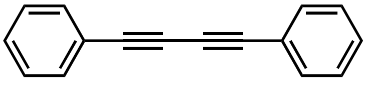

1,4-Diphenylbuta-1,3-diyne: white solid, ^{1}H NMR: δ = 7.58–7.50 (m, 4H), 7.42–7.30 (m, 6H) (ppm); ^{13}C NMR δ = 132.52, 129.24, 128.46, 121.81, 81.58, 73.92 (ppm);

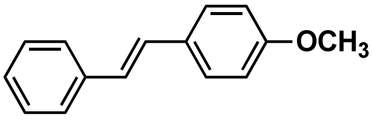

1-Methoxy-4-styrylbenzene: white solid; ^{1}H NMR: δ = 7.42 (d, 2H, *J*= 7.5 Hz), 7.37 (d, 2H, *J*= 8.5 Hz), 7.28 (t, 2H, *J*= 7.5 Hz), 7.18 (t, 1H, *J*= 6.5 Hz), 6.99 (d, 1H, *J*= 16.0 Hz), 6.89 (d, 1H, *J*= 16.5 Hz), 6.80 (d, 2H, *J*= 8.5 Hz), 3.77 (s, 3H) (ppm). ^{13}C NMR: δ = 160.5, 138.8, 130.9, 129.5, 128.0, 127.5, 127.0, 126.5, 126.0, 116.5, 57.5 (ppm).

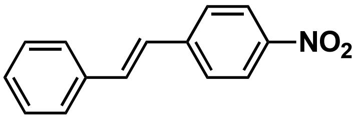

1-Nitro-4-styrylbenzene: white solid; ^{1}H NMR: δ = 8.22-8.23 (d, J = 8.4 Hz, 2H), 7.63-7.64 (d, J = 9.0 Hz, 2H), 7.55-7.57 (d, J= 7.2 Hz, 2H), 7.40-7.42 (t, J =

7.8 Hz, 2H), 7.33-7.36 (t, J= 7.8 Hz, 1H), 7.26-7.29 (d, J= 16.2 Hz, 1H), 7.16 (d, J= 16.2 Hz, 1H) (ppm). ^{13}C NMR: δ = 146.9, 144.0, 136.3, 133.4, 127.2, 127.0, 126.4, 124.3 (ppm).

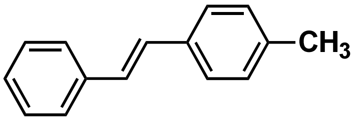

1-Methyl-4-styrylbenzene: white solid; ^{1}H NMR: δ 7.55-7.14 (9H, m), 5.43 (d, J = 16.8 Hz, 1H), 5.42 (d, J = 16.8 Hz, 1H), 2.38 (3H, s) (ppm)

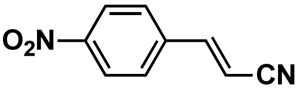

3-(4-Nitrophenyl)acrylonitrile: yellow solid ^{1} H NMR: δ = 8.06 (d, J = 8.7 Hz, 2 H), 7.57 (d, J = 8.8 Hz, 2 H), 7.20 (d, J = 16.8 Hz, 1 H), 5.84 (d, J = 16.8 Hz, 1 H) (ppm); ^{13}C NMR: δ = 148.2, 147.1, 132.4, 127.6, 127.0, 118.7, 95.6 (ppm)

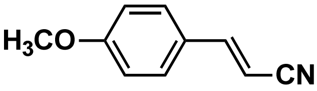

3-(4-Methoxyphenyl)acrylonitrile: yellow oil; ^{1}H NMR: δ = 7.29–7.25 (m, 3 H), 6.84 (d, J = 8.2 Hz, 2 H), 5.65 (d, J = 16.5 Hz, 1 H), 3.77 (s, 3 H) (ppm); ^{13}C NMR: δ = 162.06, 150.00, 129.05, 126.37, 118.64, 114.52, 93.40, 55.43 (ppm);

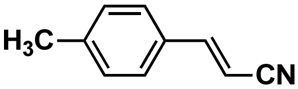

3-(4-Methylphenyl)acrylonitrile ^{1}H NMR: δ = 7.24–7.18 (m, 5 H), 5.74 (d, J = 16.7 Hz, 1 H), 2.30 (s, 3 H) (ppm); ^{13}C NMR: δ = 150.6, 141.5, 130.2, 129.6, 127.1, 118.2, 95.1, 21.4 (ppm).

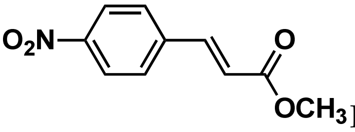

Methyl-3-(4-nitrophenyl)acrylate: white solid; ^{1}H NMR: δ = 8.25 (d, J = 8.8 Hz, 2H), 7.72 (d, J = 16.1 Hz, 1H), 7.67 (d, J = 8.7 Hz, 2H), 6.56 (d, J = 16.1 Hz, 1H), 3.84 (s, 3H) (ppm). ^{13}C NMR: δ = 166.6, 148.7, 142.1, 140.7, 128.8, 124.4, 122.3, 52.3 (ppm).

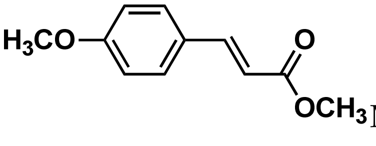

Methyl-3-(4-methoxyphenyl)acrylate: yellow solid; ^{1}H NMR: δ = 7.67 (d, J = 16 Hz, 1H), 7.50-7.47 (m, 2H), 6.94-6.90 (m, 2H), 6.33 (d, J = 16 Hz, 1H), 3.85 (s, 3H), 3.81 (s, 3H) (ppm). ^{13}C NMR: δ = 167.8, 161.4, 144.6, 129.8, 127.2, 115.3, 114.4, 55.4, 51.6 (ppm)

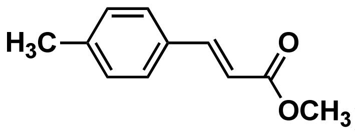

Methyl-3-(p-tolyl)acrylate: white solid; ^{1}H NMR: δ =7.69 (d, J = 16 Hz, 1H), 7.43 (d, J = 8 Hz, 2H), 7.20 (d, J = 8Hz, 2H), 6.41 (d, J = 16 Hz, 1H), 3.81(s, 3H), 2.38 (s, 3H) (ppm). ^{13}C NMR: δ = 167.6, 144.8, 140.7, 131.6, 129.6, 128.0, 116.7, 51.6, 21.4 (ppm).

Characterization:

TGA measurements were performed by placing ~2-5 mg of the sample in platinum pans and heating to 700°C at a rate of 10.0 °/min using Shimadzu DTG-60H (Shimadzu GmbH, Kyoto, Japan).

Mass spectral analyzes were carried out on the Bruker Biflex III MALDI-TOF instrument (Bruker, Billerica, MA, USA). MALDI-TOF mass spectra were measured by using a 337 nm nitrogen laser and tetracyanoquinodimethane (TCNQ) (Sigma-Aldrich, St.Louis, MO, USA) as a matrix.

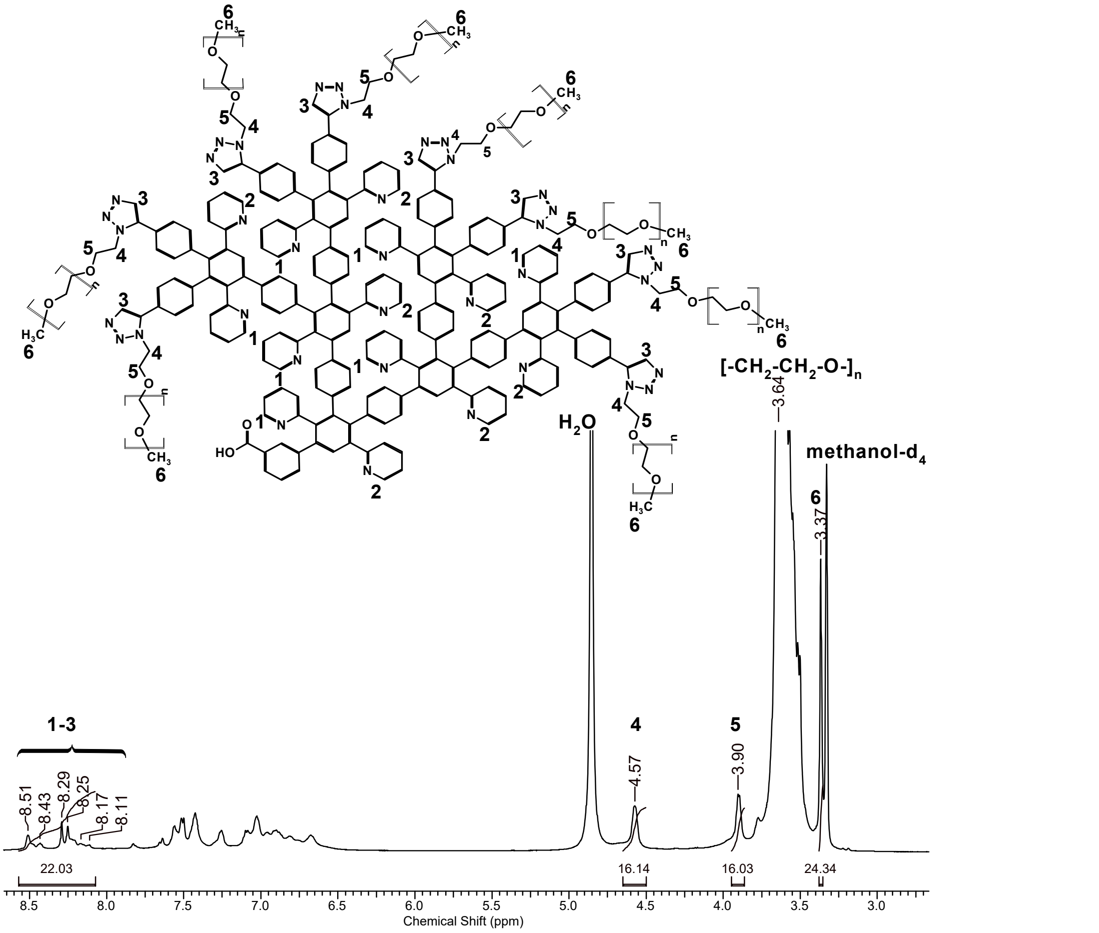

Figure S1. ^{1}H NMR of PEGylated G3.

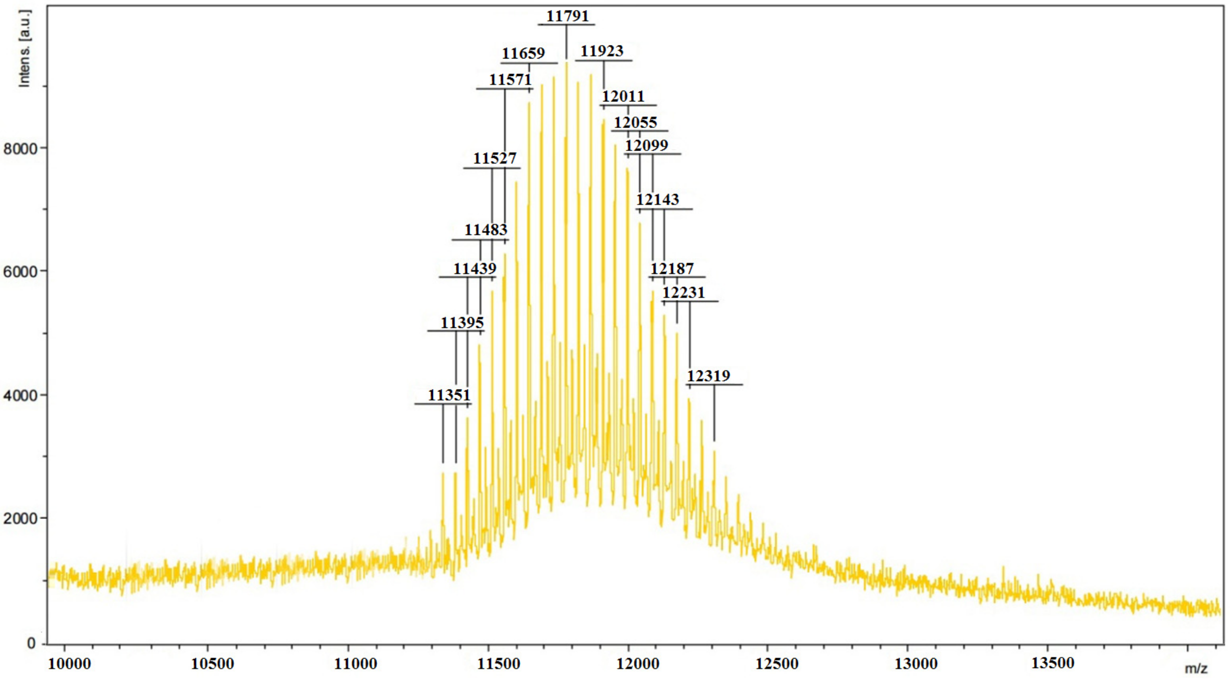

Figure S2. MALDI ToF of PEGylated G3.

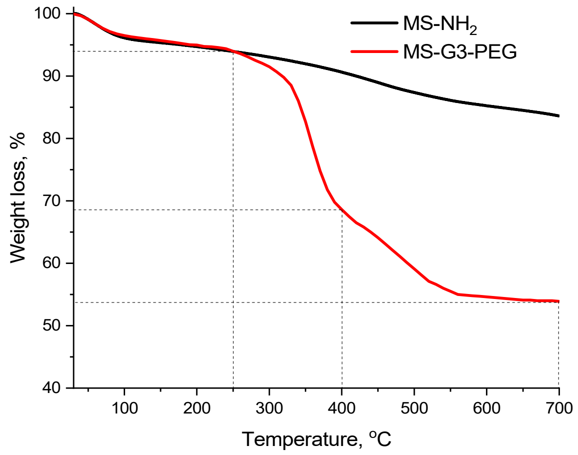

Figure S3. TGA of $MS-NH_2$ and MS-G3-PEG.

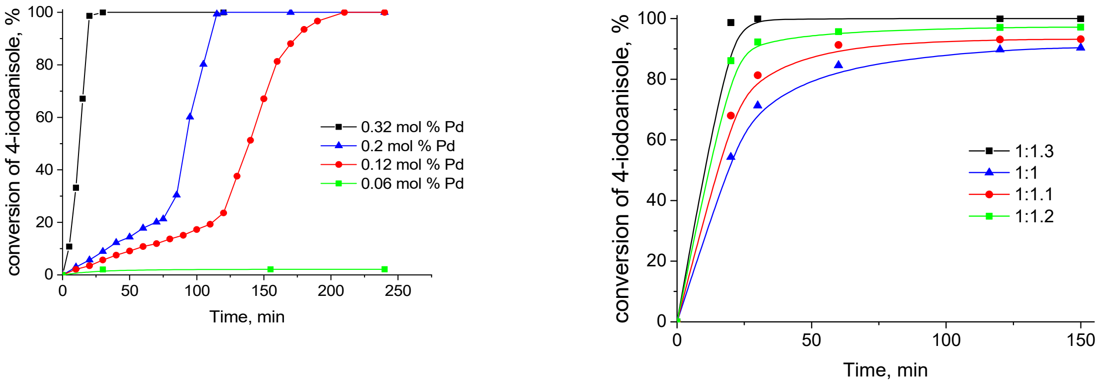

(a) (b)

Figure S4. Effect of the Pd loading (a) and the phenylacetylene molar ratio at the Pd loading of 0.32 mol % (b) on the reaction conversion.

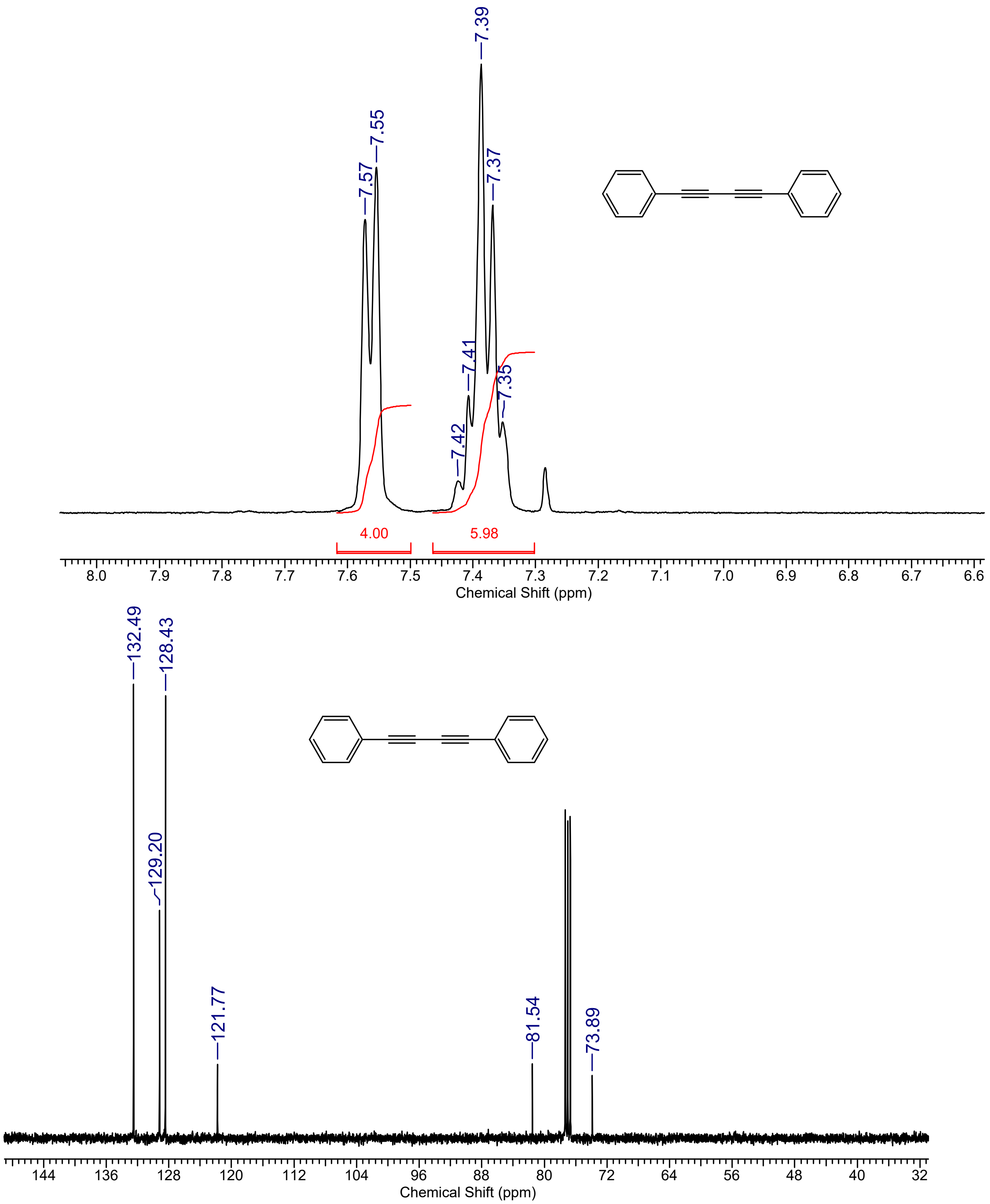

Figure S5. ^{1}H NMR and ^{13}C NMR of 1,4-diphenylbuta-1,3-diyne.

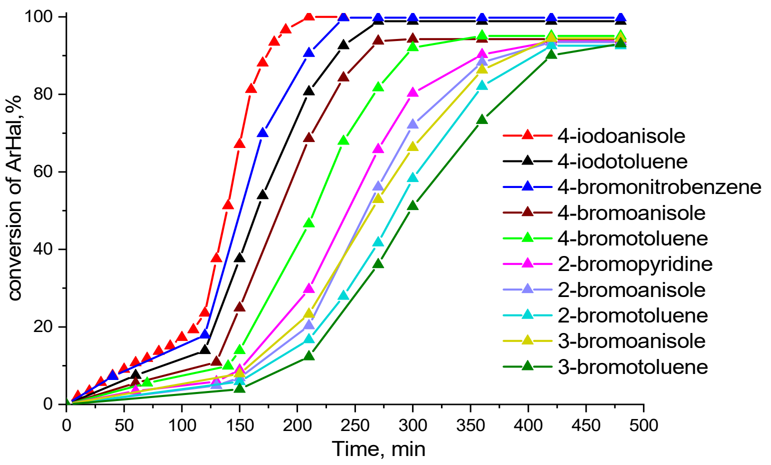

Substrate	R (mmol sub/h)
4-iodoanisole	0.81
4-iodotoluene	0.48
4-Bromonitrobenzene	0.62
4-Bromoanisole	0.44
4-bromotoluene	0.32
2-bromopyridine	0.35
2-bromoanisole	0.35
2-bromotoluene	0.28
3-bromoanisole	0.29
3-bromotoluene	0.26

Figure S6. Kinetic curves and apparent rates for intermediate conversions (after an induction period) for different substrates at the Pd loading of 0.12 mol % for the Sonogashira reaction.

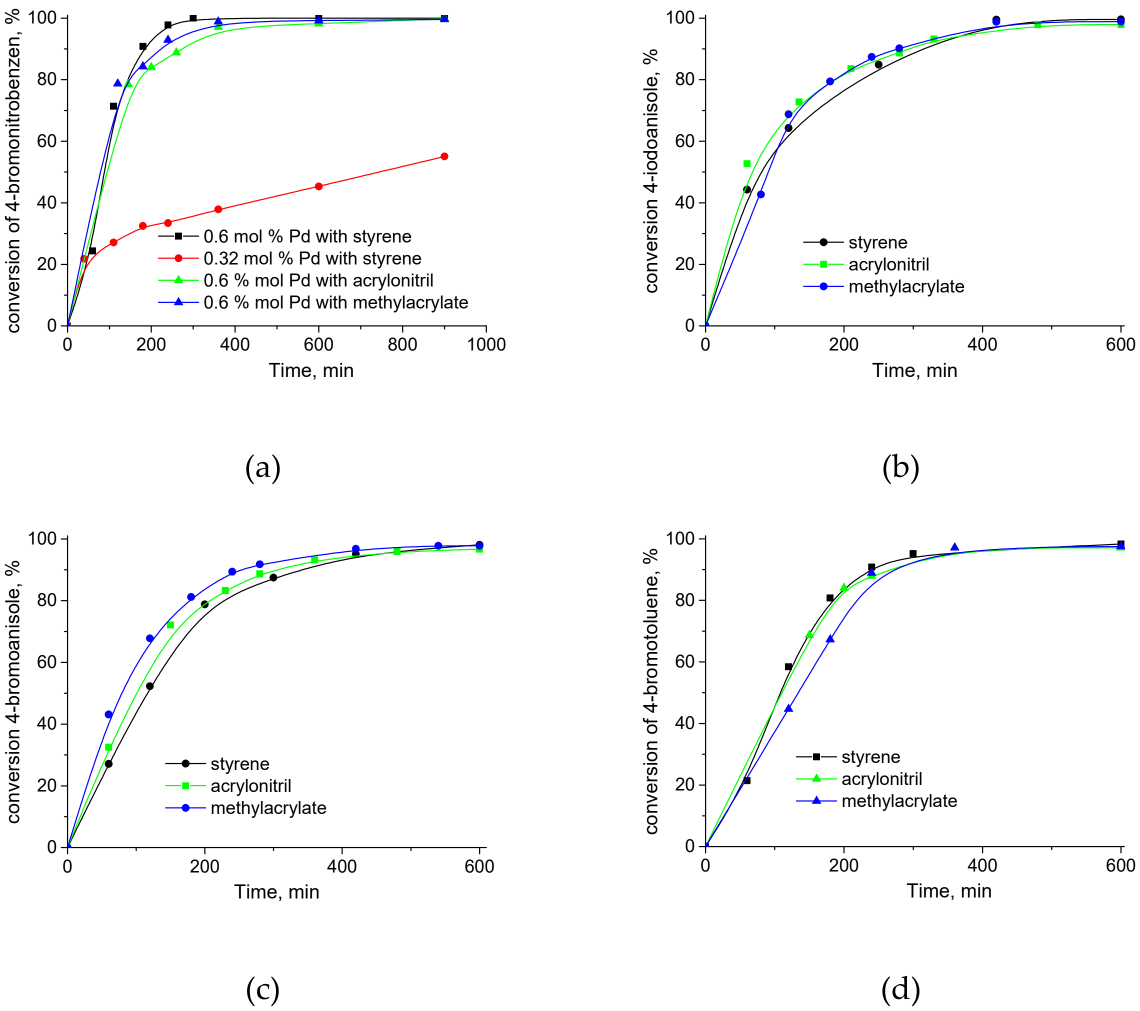

Figure S7. Kinetic curves for Heck coupling of different substrates: (a) 4-bromonitrobenzene, (b) 4-iodoanisole, (c) 4-bromoanisole, (d) 4-bromotoluene

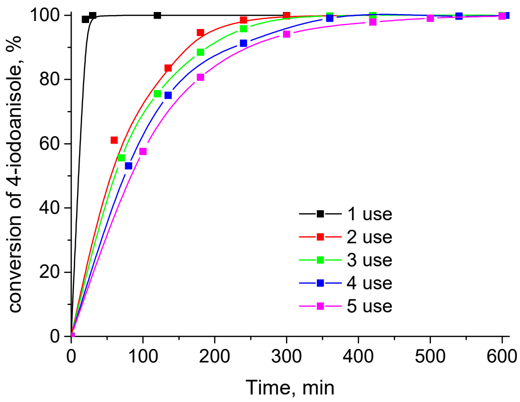

Figure S8. Kinetic curves for five consecutive catalytic cycles for Sonogashira coupling of 4-iodoanisole with phenylacetylene. In all cases, Pd loading is 0.32 mol % with respect to 4-iodoanisole.

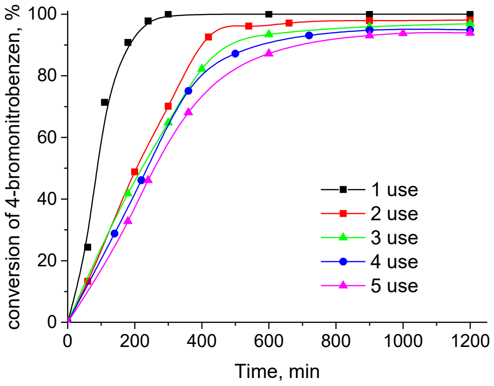

Figure S9. Kinetic curves for five consecutive catalytic cycles for Heck coupling of 4-bromonitrobenzene with styrene. In all cases, Pd loading is 0.6 mol % with respect to 4-bromonitrobenzene.

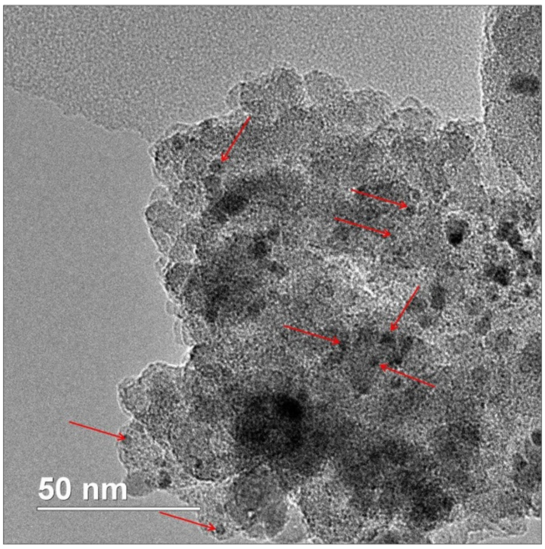

Figure S10. TEM image of MS-G3-PEG- $Pd(OAc)_2$ after the first cycle of Heck coupling.

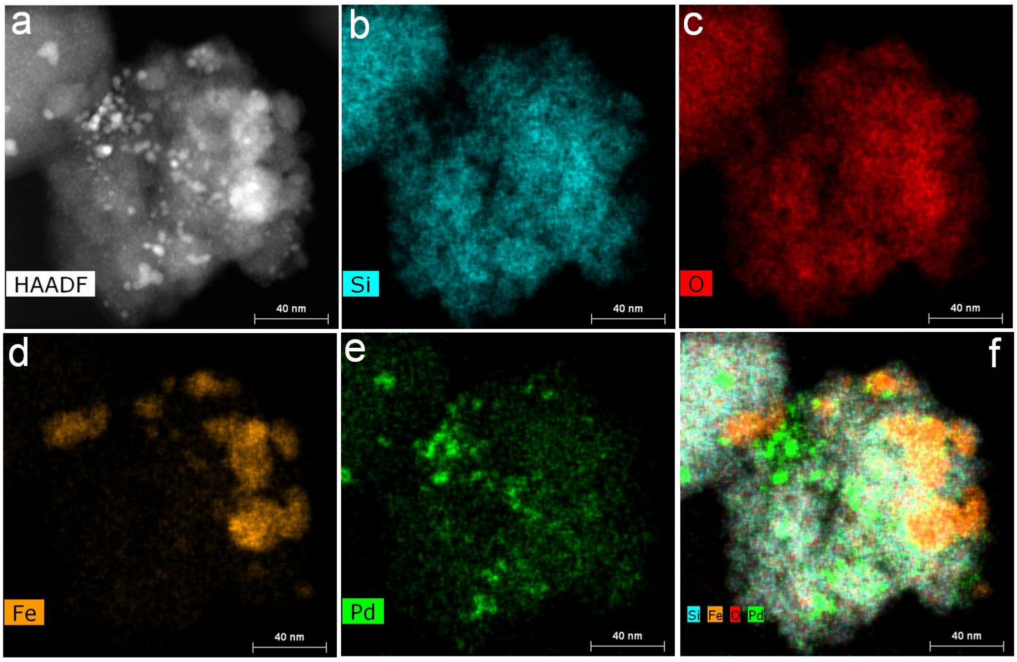

Figure S11. STEM dark-field image (a) and EDS maps for Si (b), O (c), Fe (d), and Pd (e) and their superposition (f) for MS-G3-PEG- Pd(OAc)$_2$ after the first catalytic cycle of Heck coupling.

Table S1. Fitting parameters for HR XPS Pd3d of MS-G3-PEG- Pd(OAc)$_2$.

Band	Position, eV	FWHM, eV	%Gauss	%Area	Chi Squared
1	335.8	2.34	90	22.40	1.51
2	337.73	2.57	90	37.67	
3	341.06	2.43	90	14.89	
4	342.99	2.68	90	25.04	

Table S2. Fitting parameters for HR XPS Pd3d of MS-G3-PEG- Pd(OAc)$_2$ after the first catalytic reaction.

Band	Position, eV	FWHM, eV	%Gauss	%Area	Chi Squared
1	335.12	2.26	90	28.64	1.62
2	337.59	2.86	90	31.60	
3	340.32	2.31	90	18.90	
4	342.85	2.94	90	20.86	
